# Observation of tunable chiral spin textures with nonlinear optics

**DOI:** 10.1038/s41467-026-74328-x

**Published:** 2026-06-15

**Authors:** Youqiang Huang, Tiago V. C. Antão, Adolfo O. Fumega, Mikko Turunen, Yi Zhang, Hanlin Fang, Nianze Shang, Juan C. Arias-Muñoz, Fedor Nigmatulin, Hao Hong, Andrew S. Kim, Faisal Ahmed, Hyunyong Choi, Sanshui Xiao, Kaihui Liu, Jose L. Lado, Zhipei Sun

**Affiliations:** 1 Department of Electronics and Nanoengineering, Aalto University, Espoo, Finland; 2 Department of Applied Physics, Aalto University, Espoo, Finland; 3 Department of Electrical and Photonics Engineering, Technical University of Denmark, Konges Lyngby, Denmark; 4 State Key Laboratory for Mesoscopic Physics, School of Physics, Peking University, Beijing, China; 5 Department of Physics and Astronomy, Seoul National University, Seoul, Republic of Korea

**Keywords:** Nonlinear optics, Two-dimensional materials

## Abstract

Chiral spin textures, such as spin spirals and skyrmions, are key to advancing spintronics by enabling ultrathin, energy-efficient memory, and high-density data storage and processing. However, their realization remains hindered by the scarcity of suitable host materials and the formidable experimental challenges associated with the characterization of these intricate chiral magnetic states. Here, we report the observation of tunable chiral magnetic textures in van der Waals magnet CrPS_4_ with nonlinear optics. These tunable textures exhibit strong chiral third-order nonlinear optical responses, driven by interlayer and intralayer spin couplings under varying magnetic fields and temperatures. These pronounced chiral nonlinear optical responses highlight the potency and high sensitivity of the nonlinear optical readout for probing non-collinear magnetic orders. Moreover, our findings position van der Waals magnets and their heterostructures as an exceptional platform for reconfigurable spin-photonics and spintronics, unifying optical, electrical, and magnetic properties through unique intralayer and interlayer spin coupling properties and effective spin interaction between photons and electrons.

## Abstract (web summary)

A key challenge with magnetic chirality lies in detection. Many standard probes of magnetic order, such as the magneto-optical Kerr effect, tend not to be sensitive to chiral textures. Here, Huang, Antao, and coauthors succeed in observing chiral spin texture in CrPS4 using non-linear optics.

## Introduction

Chiral spin textures, such as skyrmions and spin spirals, are of great interest in spintronics for their potential to enable compact, low-power memory and logic devices. A major bottleneck to their practical realization has been the lack of suitable material platforms with controllable and stable magnetic interactions. The emergence of two-dimensional (2D) van der Waals (vdW) magnets has addressed this challenge, offering highly tunable magnetic properties and seamless integration into layered heterostructures^1–4^. Notable examples, including CrI_3_, FePS_3_, and Cr_2_Ge_2_Te_6_, exhibit robust magnetic order down to the monolayer limit^5–11^, establishing 2D magnets as a powerful platform for studying low-dimensional magnetism. Indeed, these materials have enabled the experimental investigation of complex spin configurations and have broadened opportunities for spintronic and quantum information technologies^12–16^. Recent developments in twisted magnetic systems^17–19^ and spin-spiral multiferroics^20–23^ further highlight the potential of 2D vdW magnets to host rich and reconfigurable chiral spin textures. Nevertheless, the discovery of vdW compounds capable of supporting such states remains limited, constraining the exploration of their interplay with other emergent phenomena and impeding a full understanding of their underlying mechanisms.

These challenges are further intensified by the limitations of current techniques for characterizing complex spin textures. Although neutron powder diffraction^24,25^ is effective in detecting magnetic orders in bulk materials, it is less suitable for resolving whether the magnetic moments arrange in a collinear or noncollinear pattern in ultrathin multilayers. Optical methods, including magnetic optical Kerr effect (MOKE)^26–29^ and reflectance magneto-circular dichroism^30–32^, are sensitive to out-of-plane magnetization but fall short in probing in-plane or chiral spin configurations^17^. Nonlinear optical processes, especially third-harmonic generation (THG) circular dichroism, present a promising alternative, as shown in studies on chiral molecules^33^ and metasurfaces^34–38^. Despite this transformative potential, the application of nonlinear optics to complex spin textures remains substantially unexplored^39–42^, underscoring the need to leverage these methods for a deeper understanding of the magnetic, spintronic, and chiral nonlinear optical properties of 2D materials.

Here, we observe complex chiral magnetic orders in CrPS_4_ with nonlinear optics and propose a microscopic spin model describing their emergence due to competing interlayers magnetic couplings. Using chiral THG, we demonstrate that these frustrated magnetic states, arising from nearest- and next-nearest-interlayer exchange interactions, can be finely tuned by temperature and magnetic field. Our measurements of chiral THG reveal these tunable spin spiral phases under varying magnetic field magnitudes, showcasing chiral nonlinear optics as a powerful tool for probing non-collinear spin configurations inaccessible to conventional methods. These findings underscore the potential of 2D magnets and their heterostructures, particularly CrPS_4_, as versatile platforms for integrating optical, electrical, and magnetic chirality. The ability to merge chiral light-matter interactions with tunable chiral spin textures opens exciting avenues for advancing nonlinear reconfigurable spin-photonics, spintronics, and next-generation information storage and processing technologies.

## Results

### Tunable chiral magnetic orders in CrPS_4_

CrPS_4_ is an air-stable vdW magnet known for its A-type antiferromagnetic (AFM) behavior, where ferromagnetic (FM) layers couple antiferromagnetically along the c-axis, corresponding to the magnetic easy axis^43–46^. Owing to its AFM order, CrPS_4_ is an attractive choice for cryogenic spintronic devices^47–49^, where the absence of net magnetization eliminates stray magnetic fields that typically arise in FM counterparts^50^. However, as a consequence, external disturbances like small external magnetic fields provide a relatively weak response in AFM materials, which poses challenges in probing spin behavior. Furthermore, despite growing interest, the theoretical understanding of CrPS_4_ remains limited. While there is a consensus regarding the AFM nature of the interlayer exchange interactions along the c-axis, the potential existence of competing couplings has remained an open question.

**Fig. 1:**
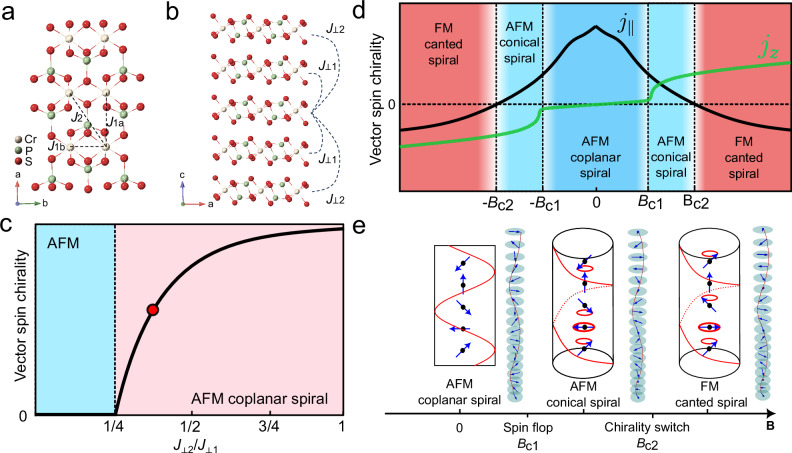
Crystalline structure, exchange interaction model, and predicted magnetic orders in CrPS_4_. **a** Schematic representation of the atomic structure of CrPS_4_ with highlighted intralayer exchange interactions: *J*_1*a*_, *J*_1*b*_, and *J*_2_. **b** Interlayer AFM exchange interactions (including nearest *J*_⊥1_ and next-nearest *J*_⊥2_ interlayer interactions), giving rise to the AFM coplanar spiral phase at zero field. **c** Schematic magnetic phase diagram as a function of the ratio of *J*_⊥2_/*J*_⊥1_. The red spot represents the theoretically calculated value of *J*_⊥2_/*J*_⊥1_ for CrPS_4_. **d** Vector spin chirality as a function of the magnetic field, captured experimentally by the THG circular dichroism. Calculated even (in-plane, *j*_∥_) and odd (out-of-plane, *j*_*z*_) components are represented in black and light green, respectively. The first critical field, ± *B*_c1_, triggers the spin-flop transition from an AFM coplanar to an AFM conical spiral, while the second, ± *B*_c2_, induces a chirality switch, marking the AFM conical to FM canted spiral transition. **e** Diagram of spin textures as a function of the magnetic field, illustrating spin-flop and spin chirality switch: AFM coplanar spiral, AFM conical spiral, and FM canted spiral.

In terms of its crystalline structure (see Fig. 1a), monolayer CrPS_4_ comprises CrS_6_ edge-sharing octahedra, forming quasi-one-dimensional chains aligned along the b-axis. These chains are interconnected along the *a*-axis through PS_4_ tetrahedra. Structurally, the quasi-one-dimensional chains define the dominant intralayer exchange pathways, which stabilize robust FM order within each vdW layer. The magnetic moments of CrPS_4_ within each layer are placed in a rectangular lattice formed by the Cr^3+^ ions. Spins within each layer are coupled to their neighbors by intralayer symmetric anisotropic FM exchange interactions (see Fig. 1a) and interlayer symmetric AFM exchange interactions (see Fig. 1b).

From first-principles density functional theory (DFT) methods (detailed in “Methods” and Supplementary materials), the first (*J*_1*a*_), second (*J*_1*b*_), and third neighbor (*J*_2_) exchange interactions within each layer (see Fig. 1a) can be estimated. These intralayer couplings lead to an FM order within each plane so that all the spins within a vdW plane remain collinear. In contrast, our DFT calculations show that a non-collinear spin spiral order between vdW planes emerges as a consequence of competition between nearest and next-nearest interlayer exchange interactions. Similar orders in other types of materials have been reported, for instance, as a consequence of spin-orbit coupling terms such as the interlayer Dzyaloshinskii-Moriya interaction^51–53^. With this picture in mind, the spin-flop transition reported in bulk CrPS_4_ stems from a metamagnetic transition associated with competing interlayer interactions, with the potential of featuring more complex phase transitions. To model this material, since the intralayer exchanges do not result in any additional frustration down to the monolayer limit, we can integrate out the degrees of freedom of individual spins within each layer, leading to a macro-spin Hamiltonian that reads

1 $${H}_{\perp }={J}_{\perp 1}\mathop{\sum }\limits_{n}{{\bf{S}}}_{n}\cdot {{\bf{S}}}_{n+1}+{J}_{\perp 2}\mathop{\sum }\limits_{n}{{\bf{S}}}_{n}\cdot {{\bf{S}}}_{n+2}+{A}_{\parallel }\mathop{\sum }\limits_{n}\left[{\left({S}_{n}^{x}\right)}^{2}+{\left({S}_{n,i}^{y}\right)}^{2}\right]$$

*A*_∥_ denotes the in-plane magnetic anisotropy. The nearest (*J*_⊥1_) and next-nearest (*J*_⊥2_) interlayer exchange interactions in CrPS_4_ (as shown in Fig. 1b) are estimated with DFT calculations indicating a ratio *J*_⊥2_/*J*_⊥1_ well above the necessary threshold for non-collinear magnetism (see Fig. 1c). As known from the theory of frustrated magnets, competing AFM interactions between the first *J*_⊥1_ and second neighbors *J*_⊥2_ in a spin-chain can stabilize a spin-spiral phase in the event that *J*_⊥2_/*J*_⊥1_ > 1/4. Such a ground state can be determined by minimizing the energy of the spin Hamiltonian, from which we can confirm the presence of a spin-spiral phase.

Rationalizing this emergent magnetic phase requires quantifying the non-collinearity of the magnetic texture, thereby characterizing both its chirality and periodicity. This can be achieved by making use of the average vector spin chirality, defined as ***j*** = 〈**S**_*n*_ × **S**_*n*+1_〉 ^54,55^. We shall see that this quantity evolves under a magnetic field in a manner compatible with the measured chiral THG response. The in-plane vector spin chirality ${j}_{\parallel }=\pm \sqrt{{j}_{x}^{2}+{j}_{y}^{2}}$, together with out-of-plane component *j*_*z*_ then serve as the order parameters to capture both in-plane chirality switches as well as spin-flop transitions. It is worth noting that commensurability effects between the number of layers in the CrPS_4_ samples and the periodicity of the spiral induced by the *J*_2⊥_/*J*_1⊥_ ratio play a substantial role in determining the magnetic order and vector spin chirality of the material (see “Methods” and Supplementary materials for details). The experimental observations from CrPS_4_ are accounted for by taking *J*_1⊥_ ≈ 0.2 meV, and *J*_2⊥_ ≈ 0.07 meV, resulting in a ratio of *J*_2⊥_/*J*_1⊥_ ≈ 0.35 (indicated by the red spot in Fig. 1c). We can therefore rationalize the effects of a magnetic field on few-layer CrPS_4_ from the perspective of the vector spin chirality as shown in Fig. 1d with estimated order parameters (i.e., *j*_∥_ and *j*_*z*_). Without a magnetic field, this material displays a spontaneous finite vector spin chirality due to the competing interactions, reflecting a chiral magnetic texture driven by the competition between *J*_⊥1_ and *J*_⊥2_. This corresponds to a coplanar spin spiral phase (see the Supplementary for more descriptions). Beyond the spin-flop critical field (*B*_c1_), the canting of spins drives a transition from the spontaneous AFM coplanar spiral phase to an AFM conical spiral phase. In this state, the spins adopt a helical arrangement, forming a screw-like structure that gives rise to an out-of-plane chiral spin texture. When the applied magnetic field exceeds *B*_c2_, a further transition occurs from the AFM conical spiral phase to the FM canted spiral phase, accompanied by a chirality switch. Figure 1e presents a schematic representation of the predicted diverse spin spiral phases, highlighting the spin-flop transition and subsequent switch in spin chirality. In the AFM conical spiral phase, adjacent spins are nearly anti-parallel, resulting in a characteristic sign for the spin cross-product **S**_*i*_ × **S**_*i*+1_. As the system transitions to the FM canted spiral phase, where spins become more aligned, this sign reverses. Consequently, the change in *j*_∥_ reflects the realignment of spins, leading to a spin chirality switch.

### Chiral THG induced by non-collinear spins

**Fig. 2:**
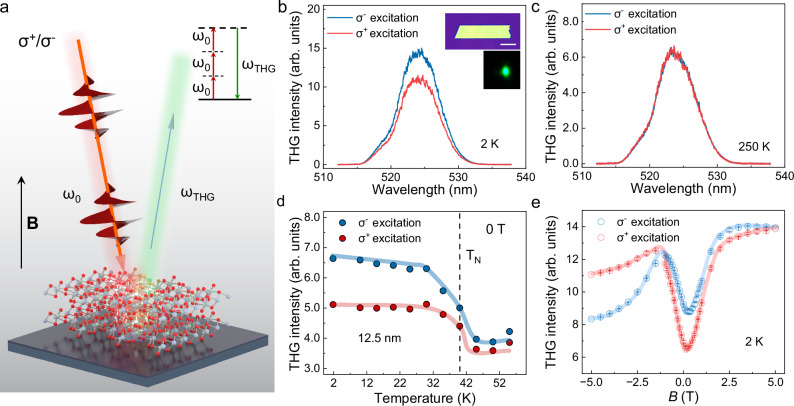
Chiral THG in CrPS_4_. **a** Illustration of chiral THG measurement. *ω*_0_: the fundamental frequency, *ω*_THG_: the frequency of THG. Inset: schematic THG process. THG spectra of a ~ 12 nm thick flake pumped with different chiralities at the temperature of (**b**) 2 K and **c** 250 K. Insets in (**b**) display the optical image of the CrPS_4_ flake and the THG beam obtained by 1575 nm laser excitation, as captured by an imaging camera. Scale bar is 5 μm. **d** Temperature-dependent THG intensity of the ~ 12 nm thick flake. T_N_ indicates the Néel temperature ~ 40 K. **e** Out-of-plane magnetic field dependence of the chiral THG intensity at the temperature of 2 K. Error bars denote the standard error of the mean, calculated from three independent measurements. Blue and red shadings visually accentuate the data trends.

In our experiments, we investigate the non-collinear spin properties of CrPS_4_ by measuring THG using a circularly polarized incident laser beam with different chiralities at normal incidence, as shown in Fig. 2a. It is noteworthy that no second-harmonic generation (SHG) is observed (Supplementary Fig. 8) within our system sensitivity limit, indicating a possible centrosymmetric crystal structure (C_2/*m*_) of CrPS_4_ reported in previous studies^56^. At the same time, from a symmetry-breaking perspective, the canted spin order can, in principle, induce SHG in the electric-dipole approximation^42,57^. The lack of an observable SHG signal from CrPS_4_ may therefore arise either from a weak SHG response or from the limited sensitivity of our current setup. Details of THG experimental methods and sample characterization are given in the method and Supplementary materials. Figure 2b displays the THG spectra from a ~ 12 nm thick CrPS_4_ flake (thickness identified in Supplementary Fig. 3), excited by a ~ 1575 nm femtosecond beam with different circular polarizations at the temperature of ~ 2 K. The output THG signal is linearly polarized, as shown in Supplementary Fig. 9. The insets show an optical image of the CrPS_4_ flake and the corresponding strong THG signal at 1575 nm laser excitation. The THG intensity generated by the left-handed circularly polarized (*σ*^−^) pump beam is higher (~ 1.4 times) than that produced by the right-handed circularly polarized (*σ*^+^) beam. In contrast, at the higher temperature of 250 K, the two THG intensities equalize (Fig. 2c). Figure 2d presents temperature-dependent THG measurements over ~2–55 K. The THG intensity decreases for both *σ*^−^ and *σ*^+^ light excitation as the temperature increases. Above ~ 40 K, the difference in THG intensity between the two chiralities becomes relatively small. The temperature of ~40 K fits well with the previously published AFM transition at the Néel temperature (T_N_ ≈ 40 K) of CrPS_4_^58^, indicating the correlation between chiral THG responses and spin properties in CrPS_4_.

To further investigate the correlation between chiral THG and spin properties in CrPS_4_, we conduct out-of-plane magnetic field-dependent chiral THG measurements, as shown in Fig. 2e. When an upward magnetic field is applied (*B* > 0 T), the THG intensities excited by both *σ*^−^ and *σ*^+^ beams increase and become nearly identical. However, under a downward magnetic field (*B* < 0 T), both THG intensities initially increase as the magnetic field rises to ~ −1.2 T, after which they begin to decrease. The THG intensity excited by the *σ*^+^ light exhibits a more rapid increase initially (red curve in Fig. 2e) until ~ −1.2 T, after which it decreases at a slower rate compared to the *σ*^−^ light excitation. The crystal does not exhibit circular birefringence, as reported previously^43^. We also confirm that the pronounced magnetic field-dependent chiral THG does not stem from the reflection circular dichroism (see Supplementary Figs. 10 and 11), but instead arises from the intrinsic spin texture of the CrPS_4_ flake, emphasizing a robust chirality-dependent nonlinear optical interaction driven by the material’s intrinsic chiral magnetic properties. In particular, the results suggest that an emergent spin spiral phase breaks spatial inversion symmetry by spontaneously generating a non-centrosymmetric magnetic structure. Further, the handedness of the spiral, captured by the vector spin chirality, introduces asymmetry in the interaction with circularly polarized light through a nonlinear process, allowing for a strong chiroptical THG response.

### Probing spin phase transition with chiral THG

To gain deeper insights into the underlying magnetic properties using chiral THG, we utilize the THG circular dichroism (THG_CD_), defined as ${{\rm{THG}}}_{{\rm{CD}}}=\frac{{I}^{-}-{I}^{+}}{{I}^{-}+{I}^{+}}$, where *I*^−^ represents the THG intensity excited by *σ*^−^ light, and *I*^+^ corresponds to the THG intensity generated by *σ*^+^ light. Using data obtained at 2 K in Fig. 2b, the calculated THG_CD_ is ~ 14.5% for the ~ 12 nm thick flake. While the THG intensity from CrPS_4_ follows the expected power-law dependence (Supplementary Fig. 12), the THG_CD_ signal shows negligible dependence on excitation power (Supplementary Fig. 13). This indicates that the observed THG_CD_ originates from intrinsic properties rather than any external light-manipulation effects. Accordingly, we fixed the average excitation power at ~ 10 mW (power density ~ 12.7 GW/m^2^) for all measurements to ensure consistency and reproducibility. We also perform THG_CD_ mapping, which confirms that THG_CD_ primarily originates from the flake rather than the substrate (see Supplementary Fig. 14). Furthermore, flipping the flake additionally offers the method of reversing its chiroptical response (see the Supplementary Fig. 15), fully confirming that the observed chiroptical response originates from the flake itself. Similar THG_CD_ signals are also observed in flakes with thicknesses ranging from ~ 5 to 80 nm (Supplementary Fig. 16), with detailed data for a ~ 5-nm-thick flake provided in Supplementary Fig. 17. Unlike MOKE and SHG, which are sensitive to net magnetization or layer-parity–dependent symmetry breaking, ^59^ THG is even allowed in centrosymmetric systems. It suggests that the observed chiral signal is governed primarily by competing near and next-near interlayer exchange interactions that persist in multilayer CrPS_4_.

**Fig. 3:**
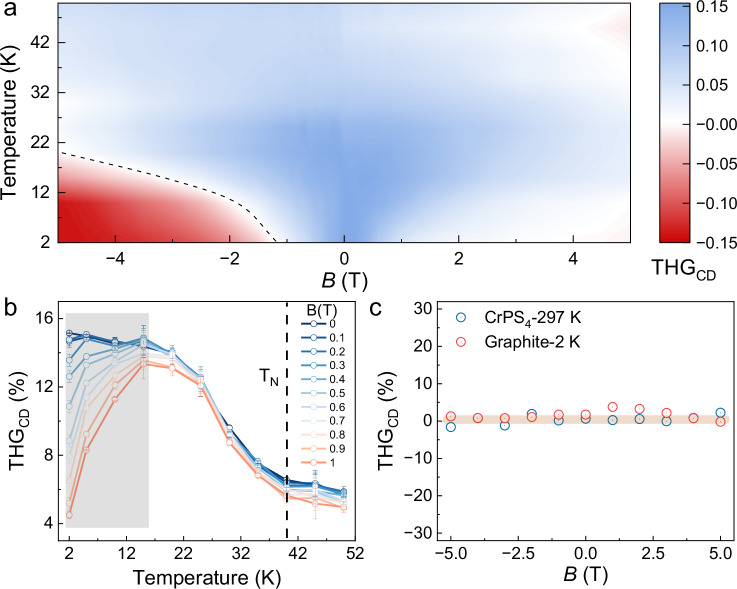
Magnetic field and temperature-dependent THG circular dichroism in CrPS_4_. **a** Mapping results in the ~ 12 nm thick CrPS_4_ flake. Dotted lines are plotted at points where THG_CD_ equals zero to guide the eye, and they denote where the optical chirality response switch occurs. **b** Temperature-dependent THG_CD_ under the *B* field of 0 to 1 T. Gray shading denotes the major impact region. Error bars denote the standard error of the mean, calculated from three independent measurements. **c** Magnetic field dependent THG_CD_ from the same CrPS_4_ flake at 297 K and a graphite reference sample at 2 K. Origin-shaded area denotes where THG_CD_ equals zero to guide the eye.

The THG_CD_ of the ~12 nm thick flake is further measured as a function of temperature (2–50 K) and magnetic field (−5 to 5 T), as shown in Fig. 3a. The temperature dependence of THG_CD_ up to 250 K is presented in Supplementary Fig. 18. Notably, the THG_CD_ values range from ~ −0.14 to 0.15 for the flake across the explored temperature and magnetic field range. We emphasize that the interpretation of the chiral THG signal does not depend on perfect inversion symmetry of the crystal structure. While even if weak structural inversion symmetry breaking cannot be completely excluded, any resulting structural chirality would be expected to exhibit weak temperature dependence. Instead, the THG_CD_ signal shows a pronounced enhancement in the magnetically ordered phase and vanishes well at higher temperatures. This behavior supports a dominant magnetic origin of the THG_CD_ response.

Under an upward magnetic field (*B* > 0 T), THG_CD_ decreases and approaches zero. Conversely, when a downward magnetic field is applied (*B* < 0 T), the flake exhibits sign changes in THG_CD_ (highlighted by dotted lines in Fig. 3a). For instance, at 2 K, THG_CD_ transitions from positive to negative at *B* ≈ −1.2 T, which aligns with the turning point observed in Fig. 2e at *B* ≈ −1.2 T. When an in-plane magnetic field of ± 2.5 T is applied, the THG_CD_ shows no significant change. This result indicates that the nonlinear optical response is highly sensitive to the magnetic field along the easy axis (*c*-axis), whereas it is insensitive to the applied fields along the in-plane hard axis(Supplementary Fig. 19).

To emphasize the influence of the magnetic field, Fig. 3b provides detailed plots of the temperature and magnetic field dependence of THG_CD_. The results reveal that the magnetic field significantly impacts the THG_CD_ below ~15 K (marked in the gray-shaded area). As the temperature approaches the Néel temperature (T_N_, ~ 40 K), the THG_CD_ signal diminishes rapidly at different magnetic fields and becomes relatively constant for temperatures above T_N_. While long-range AFM order disappears above the Néel temperature, short-range magnetic correlations and spin fluctuations might persist and contribute to a weak THG_CD_ signal. This residual response is strongly suppressed with increasing temperature and vanishes completely at higher temperatures (e.g., 250 K), consistent with the thermal destruction of all magnetic correlations. We also measured THG_CD_ from CrPS_4_ at room temperature and from graphite at 2 K. In both cases, no significant changes were observed under different magnetic fields (Fig. 3c). These findings demonstrate that the chiral third-order nonlinear optical response of CrPS_4_ is strongly influenced by temperature and external magnetic fields, a behavior uniquely associated with the chiral spin textures in the material. To further rationalize this connection, a Landau–Ginzburg treatment of the emergent third harmonic polarization under circularly polarized light and in the presence of the spin chirality axial vector is performed in the Supplementary, providing a phenomenological connection between the magnetic order of the material and the THG_CD_. Such a treatment avoids a microscopic derivation of such an expression for the THG_CD_ and associated third-order polarization involving complex multipolar contributions^60–62^, while keeping the essential features of the problem based on symmetry considerations, namely the presence of C_2/*m*_ in-plane symmetry. In particular, considering the broken time-reversal and inversion symmetries of the magnetic ground state of CrPS_4_, and writing out the third-order polarization responses that couple to the vector spin chirality **j**, we can arrive at

2 $${{\rm{THG}}}_{{\rm{CD}}}=\frac{{2}{ℑ} \left({\alpha }{*}\beta \right){j}_{z}+2ℑ \left({\eta }{*}\beta \right)k{j}_{z}^{2}+ℑ\left(\beta {\gamma }{*}\right)k{j}_{\parallel }^{2}}{| \alpha+\eta k{j}_{z}{| }^{2}+| \beta {| }^{2}\left({j}_{z}^{2}+\frac{1}{2}{j}_{\parallel }^{2}\right)+| \gamma {| }^{2}{k}^{2}{j}_{\parallel }^{2}},$$

where *α*, *β*, *γ*, and *η* are Landau–Ginzburg expansion coefficients and *k* stands in for the magnitude of the wave-vector. In particular, *α* precedes the simplest possible third order term **E**(**E** ⋅ **E**), *β* precedes a symmetry allowed term **j** × **E**, while *γ* and *η* precede terms that include **j** ⋅ **k** and **j** × **k**, respectively. Despite the slightly more complex form of this expression, it is clear that the odd part of the THG_CD_ as a function of magnetic field will be dominated by *j*_*z*_, whereas the even part is dominated by *j*_∥_, which justifies our choice of order parameters for the magnetic transitions, as detailed in the following discussion.

### Correlating tunable chiral spin textures with nonlinear optics

**Fig. 4:**
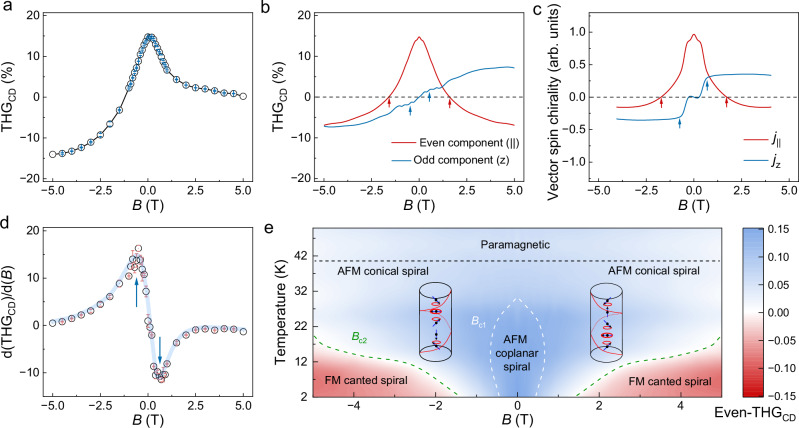
Correlation between chiral THG measurement with the spin-feather structures in CrPS_4_. **a** Experimental results of THG_CD_ for the ~ 12 nm thick sample vs. *B* field at 2 K. Error bars denote the standard error of the mean, calculated from three independent measurements. **b** Decomposition of the THG_CD_ into even and odd components. Blue arrows mark changes in the behavior of the odd component, consistent with the predicted spin-flop transition at field *B*_c1_, while red arrows mark sign switches in the even component of THG_CD_ at critical field *B*_c2_. **c** Predicted in-plane (*j*_∥_) and out-of-plane (*j*_*z*_) vector spin chirality averaged over an 18-layer (i.e., ~ 12 nm) flake. A clear analogy is verified between the odd and even components of the THG_CD_ and the vector spin chiralities, which serve here as a measure of the flake’s chirality and non-collinearity. **d** Derivative of the THG_CD_ data shown in (**a**). The blue arrows indicate a rapid change in THG_CD_, which is consistent with the theory and closely aligns with the previously reported spin-flop transition in the bulk material ^63^. Error bars denote the standard error of the mean, calculated from three independent measurements. Blue shading highlights the trend to guide the eye. **e** Mapping of even components of THG_CD_ as a function of *B* field and temperature. The dashed white and green lines correspond to the critical field ± *B*_c1_ and ± *B*_c2_, respectively. The black dashed line denotes the Néel temperature.

We now draw the correlations between the THG measurement and the chiral spin structures in the case of CrPS_4_. We first plot the THG_CD_ as a function of the magnetic field at 2 K in Fig. 4a. When an upward magnetic field is applied (*B* > 0 T), the THG_CD_ signal decreases and becomes close to 0. However, under a downward magnetic field (*B* < 0 T), the THG_CD_ starts to drop with the magnetic field and becomes zero at ~ −1.2 T, and continues to drop to ~ −14% at −5 T. These results suggest an optical chirality response switch under a magnetic field. The asymmetrical curve regarding the experimentally applied magnetic field corresponds to the mixing of contributions stemming from in-plane vector spin chirality *j*_∥_ and out-of-plane component *j*_*z*_. From the perspective of the magnetic order, the in-plane vector spin chirality together with the out-of-plane component serve as the order parameters to capture both the spin chirality switch as well as the spin-flop transition. Both quantities can be experimentally accessed by examining the even and odd components of the chiroptical THG response of the material, as seen in Fig. 4a–c. For better comparison, we extract the even and odd components of the chiroptical response of CrPS_4_ in Fig. 4b. Figure 4c shows the calculation of the in-plane, out-of-plane vector spin chirality under a magnetic field.

Figure 4b, c showcase significant correlations between the extracted even and odd components of THG_CD_ and the spin chirality. A direct comparison of the curves plotted in Fig. 4b, c suggest that the chiroptical THG response can be well captured by the behavior of the vector spin chirality (see Supplementary for theory details from the Landau–Ginzburg perspective). The behavior of ***j*** can be understood as follows: As the external magnetic field is increased from *B* = 0 T, the oscillations in spin along the different layers in the *c*-axis direction tend to align preferentially with the field. Without an external field, the spins oscillate along the layers, reaching the maximum and minimum allowed values for the projection in the out-of-plane direction *S*^*z*^ = ± 3/2 and all lie in a single plane. The magnetic field results in an effective decrease in non-collinearity, which is quantified by an overall decrease in the in-plane vector spin chirality *j*_∥_ caused by a rearrangement of the oscillations. This causes an overall preference towards either the positive or negative direction along the *c*-axis, depending on the direction of the applied magnetic field. On the other hand, *j*_*z*_ remains close to zero in this first regime, while the decrease in *j*_∥_ is accompanied by a subtle magnetization increase. At a value of magnetic field *B*_c1_ ≈ 0.49 T, a sharp increase in the *j*_*z*_ reflects the occurrence of the spin-flop transition. This is accompanied by a sharp increase in *j*_*z*_ to a finite value, which can be positive or negative depending on the field direction. This behavior, since the spin configuration no longer attains a maximum and minimum projection *S*^*z*^ = ± 3/2, is no longer coplanar. Instead, it stabilizes a sort of AFM conical spiral phase, with *S*^*z*^ oscillating symmetrically around a finite positive value. Continuously increasing the magnetic field magnitude above the spin-flop cants all the spins towards the same direction with the average sign of the in-plane vector spin chirality *j*_∥_ being switched at a critical magnetic field *B*_c2_ ≈ 1.5 T. For much larger fields than those considered here, the canting culminates in an FM phase, with the Zeeman coupling dominating over all other sources of magnetic frustration. Thus, two magnetic phase transitions are observed, the first corresponding to a fast decrease in *j*_∥_ and a sharp increase in *j*_*z*_, and the second to a sign switch in *j*_∥_. To gain more insight into these transitions, we can also compute the derivative of the THG_CD_ data relative to the magnetic field (Fig. 4d) at 2 K. Two distinct transition points (marked by blue arrows), indicating fast changes in the magnetic order, are observed at approximately ± 0.5 T, which align closely with the spin-flop transition at around 0.55 T in bulk CrPS_4_ materials at low temperatures ^63^, although the precise values vary depending on the specific crystals studied. Figure 4e illustrates the various magnetic phases at finite temperature with our experimental data, including the paramagnetic state, AFM coplanar spiral, AFM conical spiral, and FM canted spiral. Notably, the FM canted spiral phase is realized when the even component of THG_CD_ exhibits a sign change. This phase remains stable beyond the critical field *B*_c2_; however, as the temperature increases, thermal fluctuations destabilize the spin configuration, necessitating a stronger magnetic field to sustain the FM canted spiral state. These results demonstrate that THG_CD_ is sensitive to magnetic phase transitions, providing insights into the underlying tunable spin behavior. Although our developed technology in the work does not directly image the domain, establishing a direct correlation between spin chirality and nonlinear optical responses is highly desirable. To address this, our THG-based approaches have strong potential to serve as sensitive in situ or near-field probes of complex magnetic structures in low-dimensional magnets. For clarity, we compare THG and other commonly used magnetic probes in Supplementary Table 1. We emphasize that THG_CD_ does not directly determine the magnetic structure, and direct structural evidence is still needed to better understand the underlying physics. We also note that previous theoretical studies have shown that spin chirality can appear in transport and optical responses^42,64–66^. Extending such measurements to CrPS_4_ would therefore be very helpful for understanding the role of chiral magnetic correlations in this system. Nevertheless, our results suggest that THG-based techniques could be a useful way to probe complex magnetic states.

## Discussion

In conclusion, we have observed spontaneous chiral AFM spin textures in the vdW magnet CrPS_4_ through the correlation between its optical and magnetic chiral interaction. Our findings demonstrate that CrPS_4_ supports a diverse range of chiral magnetic structures, including AFM coplanar spiral, AFM conical, and FM canted spin spirals. These chiral behaviors are highly tunable, governed by the interplay between the interlayer and intralayer spin coupling under applied magnetic fields. Moreover, our chiral THG experiments unveil the evolution of chirality driven by complex spin spiral structures under varying magnetic fields and temperatures. This study demonstrates a highly sensitive and efficient method for probing spin chirality, shedding light on intricate spin coupling interactions. Beyond its significance as a powerful experimental tool for fundamental research, our approach also accelerates technological advancements in spin-photonics and spintronics. Our findings establish CrPS_4_ and its heterostructures as a unique platform for exploring spin chirality in emerging 2D magnets, where leveraging advanced 2D device fabrication techniques and stacking engineering, the interplay between intra- and interlayer exchange interactions, magnetic order, and light-matter coupling could be effectively manipulated to enable innovative applications. Ultimately, the optical probing of spin chirality provides a new strategy to understand chiral magnetic phenomena, paving the way to unlock opportunities for the development of high-speed, energy-efficient chiral devices.

## Methods

### Sample preparation and characterization

Flux zone-grown CrPS_4_ crystals are purchased from 2D Semiconductor. CrPS_4_ flakes are mechanically exfoliated by the dry transfer technique using a PDMS stamp to silicon chips with 285 nm thick SiO_2_. The thickness of the samples is characterized using atomic force microscopy with a Dimension Icon system from Bruker. The Raman spectrum is measured by a WITEC alpha 300 RA+ system at room temperature. A 532 nm continuous wave laser is focused by a 100× CF Plan Nikon objective (NA = 0.95), and the reflected Raman signal is collected by the same objective and sent to a spectrometer.

### Chiral THG measurement

Circular polarization-dependent optical THG measurements at different temperatures are carried out using a custom-built confocal optical microscope, operating in reflection geometry (see Supplementary Fig. 2 for details). The sample is mounted into a closed-loop cryostat (attoDRY 2100) equipped with superconducting magnets. The femtosecond laser at 1575 nm (FemtoFiber smart 780) is focused onto the sample by a low-temperature objective from Attocube (LT-APO/Telecom, NA = 0.8). A quarter-wave plate mounted on a motorized rotation stage (KPRM1E/M) is employed to rotate the chirality of the pump light. The reflected THG signal is collected by the same objective. A dichroic mirror with negligible polarization dependence is utilized to separate the signal. Then, the signal is further coupled into a multimode fiber to an Andor Shamrock 750 spectrograph equipped with an electron-multiplying charge-coupled device (Newton 970).

### Theoretical model and calculations

CrPS_4_ realizes a spin-3/2 Heisenberg model, with an easy axis associated with a positive in-plane single-ion anisotropy. The Hamiltonian for such a system in the presence of an external out-of-plane magnetic field takes the form

3 $$H=	 -\mathop{\sum }\limits_{i,j,n}{J}_{i,j}{{\bf{S}}}_{n,i}\cdot {{\bf{S}}}_{n,j}+\mathop{\sum }\limits_{n,m,i}{J}_{\perp n,m}{{\bf{S}}}_{n,i}\cdot {{\bf{S}}}_{m,i}+{A}_{\parallel }\mathop{\sum }\limits_{n,i}\left[{\left({S}_{n,i}^{x}\right)}^{2}+{\left({S}_{n,i}^{y}\right)}^{2}\right] \\ 	+g{\mu }_{B}B\mathop{\sum }\limits_{i,n}{S}_{n,i}^{z}$$

Here, the indices *i* and *j* run over each in-plane spins whereas *n* and *m* run over different layers. As such, *J*_*i*,*j*_ represents the in-plane exchange interaction between sites *i* and *j*. On the other hand, *J*_⊥*n*,*m*_ accounts for out-of-plane interlayer exchange interactions promoting an out-of-plane AFM order.

To quantitatively account for the experimentally observed chiroptical measurements, we extend the previous works to also account for the next-nearest interlayer interactions as well as include the effects of intralayer anisotropic exchanges.

In particular, we include the effect of intralayer exchange interactions up to third nearest neighbors in few-layer CrPS_4_ (*J*_1*a*_, *J*_1*b*_, and *J*_2_ in Fig. 1a), and compute the magnetic ground state for a rectangular lattice of Cr^3+^ ions.

4 $${H}_{\parallel }^{(n)}=\,\, 	 {J}_{1a}\mathop{\sum }\limits_{\langle i,j\rangle }{{\bf{S}}}_{n,i}\cdot {{\bf{S}}}_{n,j}+{J}_{1b}\mathop{\sum }\limits_{\langle \langle i,j\rangle \rangle }{{\bf{S}}}_{n,i}\cdot {{\bf{S}}}_{n,j}+{J}_{2}\mathop{\sum }\limits_{\langle \langle \langle i,j\rangle \rangle \rangle }{{\bf{S}}}_{n,i}\cdot {{\bf{S}}}_{n,j} \\ 	+{A}_{\parallel }\mathop{\sum }\limits_{i}\left[{\left({S}_{n,i}^{x}\right)}^{2}+{\left({S}_{n,i}^{y}\right)}^{2}\right]$$

where ${H}_{\parallel }^{(n)}$ stands for the intralayer Hamiltonian of the *n*th layer, such that the total Hamiltonian is $H={\sum }_{n}{H}_{\parallel }^{(n)}+{H}_{\perp}$. The term *H*_⊥_ corresponds to the interlayer component of the Hamiltonian for few-layer CrPS_4_. Taking into account exchange couplings between the nearest and next-nearest layers, it reads

5 $${H}_{\perp }=\,\, {J}_{\perp 1}\mathop{\sum }\limits_{i,n}{{\bf{S}}}_{n,i}\cdot {{\bf{S}}}_{n+1,i}+{J}_{\perp 2}\mathop{\sum }\limits_{i,n}{{\bf{S}}}_{n,i}\cdot {{\bf{S}}}_{n+2,i}$$

With this Heisenberg model, the different magnetic transitions in few layer CrPS_4_ are directly captured. In particular, in order to estimate the values, including the effects of the intralayer and interlayer interactions on the spin ground state, we have performed ab initio DFT calculations with the all-electron full-potential linearized augmented-plane-wave method as implemented in the Elk code^67^.

## Supplementary information

Supplementary Information

Peer Review file

## Source data

Source Data

## Data Availability

The data that support the findings of this study are provided as source data files with the paper. All data is available from the corresponding authors upon request. Source data are provided with this paper.
