# Supplementary material for: Observation of tunable chiral spin textures with nonlinear optics
Source: Nat Commun. 2026 Jun 15;17:7576. doi: 10.1038/s41467-026-74328-x (PMC13415826; doi:10.1038/s41467-026-74328-x)
Supplement: Supplementary file 1 — Supplementary Information [file 41467_2026_74328_MOESM1_ESM.pdf]

Supplementary Information for Observation of tunable chiral spin textures with nonlinear optics

Youqiang Huang[1†], Tiago V. C. Antão[2†], Adolfo O. Fumega[2], Mikko Turunen[1], Yi Zhang[1], Hanlin Fang[3], Nianze Shang[1], Juan C. Arias-Muñoz[1], Fedor Nigmatulin[1], Hao Hong[4], Andrew S. Kim[5], Faisal Ahmed[1], Hyunyong Choi[5], Sanshui Xiao[3], Kaihui Liu[4], Jose L. Lado[2*], Zhipei Sun[1*]

[1] Department of Electronics and Nanoengineering, Aalto University, Espoo, Finland.

[2]Department of Applied Physics, Aalto University, Espoo, Finland.

[3]Department of Electrical and Photonics Engineering, Technical University of Denmark, Konges Lyngby, Denmark.

[4]State Key Laboratory for Mesoscopic Physics, School of Physics, Peking University, Beijing, China.

[5]Department of Physics and Astronomy, Seoul National University, Seoul, 08826, Republic of Korea.

[*]Corresponding authors: zhipei.sun@aalto.fi, jose.lado@aalto.fi

[†]These authors contributed equally to this work.

Supplementary Note 1. Materials and Methods

Details on the spin model and ground state calculation method

$CrPS_4$ realizes a spin-$3/2$ Heisenberg model, with an easy axis associated with a positive in-plane single-ion anisotropy. The Hamiltonian for such a system in the presence of an external out-of-plane magnetic field takes the form

$$H = -\sum_{i,j,n} J_{i,j} \mathbf{S}_{n,i} \cdot \mathbf{S}_{n,j} + \sum_{n,m,i} J_{\perp n,m} \mathbf{S}_{n,i} \cdot \mathbf{S}_{m,i} + A_{\parallel} \sum_{n,i} \left[\left(S^x_{n,i} \right)^2 + \left(S^y_{n,i} \right)^2 \right] + g\mu_B B \sum_{i,n} S^z_{n,i} \tag{1}$$

Here, the indices i and j run over each in-plane spins whereas n and m run over different layers. As such, $J_{i,j}$ represents the in-plane exchange interaction between sites i and j. On the other hand, $J_{\perp n,m}$ accounts for out-of-plane interlayer exchange interactions promoting an out-of-plane antiferromagnetic order.

For bulk $CrPS_4$, previous studies consider either isotropic or anisotropic in-plane exchange interactions, but crucially restrict interlayer exchange interactions to the nearest layers, leading to the reported A-type antiferromagnetism observed in bulk samples. The single-ion anisotropy term $A_{\parallel}$ promotes an out-of-plane spin-configuration, with spins alternating between “up” and “down” orientations, along the z (out-of-plane) direction. For a bulk sample, the application of a small magnetic field on the order of 0.7 T [1] then leads to an out-of-plane to in-plane spin-flop collinear magnetic phase transition. This can be captured in the atomistic simulations by the effects of a Zeeman term B in the model above, where $g = 2$ is the gyromagnetic factor and μ_B is the Bohr magneton. The Zeeman term B competes with the single-ion anisotropy $A_{\parallel}$, which leads to a transition from the alternating “up”-“down” configuration, to an alternating order where spins align mostly in-plane at the critical flop value B_{c1}. This transition has been experimentally measured as a sudden increase in out-of-plane magnetization.

The modeling of the magnetic behavior of few layer $CrPS_4$ is also well captured by a Heisenberg model of the previous form. To quantitatively account for the experimentally observed chiroptical measurements, we extend the previous works to also account for the next nearest interlayer

interactions as well as include the effects of intralayer anisotropic exchanges.

In particular, we include the effect of intralayer exchange interactions up to third nearest neighbors in few-layer $CrPS_4$ (J_{1a}, J_{1b}, and J_2 in Fig. 1a of the main text), and compute the magnetic ground state for a rectangular lattice of Cr^{3+} ions.

$$
\begin{aligned}
H_{\parallel}^{(n)} =& J_{1a} \sum_{\langle i,j \rangle} \mathbf{S}_{n,i} \cdot \mathbf{S}_{n,j} + J_{1b} \sum_{\langle\langle i,j \rangle\rangle} \mathbf{S}_{n,i} \cdot \mathbf{S}_{n,j} \\
&+ J_2 \sum_{\langle\langle\langle i,j \rangle\rangle\rangle} \mathbf{S}_{n,i} \cdot \mathbf{S}_{n,j} + A_{\parallel} \sum_{i} \left[\left(S_{n,i}^{x} \right)^2 + \left(S_{n,i}^{y} \right)^2 \right]
\end{aligned} \tag{2}
$$

where $H_{\parallel}^{(n)}$ stands for the intralayer Hamiltonian of the nth layer, such that the total Hamiltonian is $H = \sum_n H_{\parallel}^{(n)} + H_{\perp}$. The term $H_{\perp}$ corresponds to the interlayer component of the Hamiltonian for few-layer $CrPS_4$. Taking into account exchange couplings between the nearest and next-nearest layers, it reads

$$
H_{\perp} = J_{\perp 1} \sum_{i,n} \mathbf{S}_{n,i} \cdot \mathbf{S}_{n+1,i} + J_{\perp 2} \sum_{i,n} \mathbf{S}_{n,i} \cdot \mathbf{S}_{n+2,i} \tag{3}
$$

With this Heisenberg model, the different magnetic transitions in few layer $CrPS_4$ are directly captured. In particular, in order to estimate the values, including the effects of the intralayer and interlayer interactions on the spin ground state, we have performed *ab initio* Density Functional Theory (DFT) calculations with the all-electron full-potential linearized augmented-plane-wave method as implemented in the Elk code [2]. We have used the LDA+U exchange-correlation function. The results presented are converged with respect to all the parameters. In particular, a $4 \times 8 \times 1$ k-mesh, $R_{kmax} = 7$. For intralayer interactions, our calculations yield $J_{1a} \approx 0.55$ meV, $J_{1b} \approx -2.41$ meV and $J_2 \approx -1.19$ meV. We observe no significant dependence with the chosen U (Figure 1). Using these values, it is then possible to estimate the spin ground state of a single layer or a set of antiferromagnetically coupled layers.

The lowest state configuration of the previous classical Hamiltonian can be obtained by the exact minimization of the spin configuration. To ensure reliability, parameters are initialized randomly, and the minimization process is repeated approximately 200 times, with the configuration yielding the lowest energy chosen as the final result. We confirm that different random initializations consistently produce equivalent ground state configurations, but it is often the case that for zero

field, one has distinct possibilities for the ground state. Namely, in the absence of any anisotropy, the spin ground state corresponds to a coplanar spiral with an arbitrary plane of propagation.

We find, that the intralayer exchange interactions as predicted from DFT calculations are insufficient to cause any sort of spin non-collinearity, highlighting that the spin non-collinearity stems from the competing interlayer interactions. These can also be predicted from DFT calculations, for which we obtain $J_{\perp 1} \approx 0.293$ meV and $J_{\perp 2} \approx 0.142$ meV, such that their ratio is $J_{\perp 2}/J_{\perp 1} \approx 0.45$, which is well above the theoretical critical value of 0.25 necessary for the occurrence of an out-of-plane spin spiral phase. It is worth noting that exact values of the exchange couplings in vdW materials depend on the details of the DFT methodology and the potential inclusion of DFT+U corrections [3–5], which will thus affect the value of the ratio $J_{\perp 2}/J_{\perp 1}$. Beyond the potential changes in the ratio due to the DFT methodology, our first principles results demonstrate the existence of competing interlayer exchange interactions leading to the emergence of non-collinear phases in few layer $CrPS_4$. Therefore, to quantitatively describe the experimental phenomenology, we take $J_{\perp 2}/J_{\perp 1} \approx 0.35$ for direct comparisons between the classical Heisenberg model and the experiment.

Due to the dominant value of intralayer exchanges, the lowest state configuration in the multilayer will feature a state where within each layer, every spin is aligned. This intralayer collinearity allows us to use of a macro-spin approach to describe the ground state of the magnet. In practice, each macro-spin represents a layer, and the whole few-layer system can be described by a few-site macro-spin chain with Hamiltonian

$$\begin{aligned} H_\perp =& J_{\perp 1} \sum_n \mathbf{S}_n \cdot \mathbf{S}_{n+1} + J_{\perp 2} \sum_n \mathbf{S}_n \cdot \mathbf{S}_{n+2} \\ &+ A_\parallel \sum_i \left[\left(S^x_{n,i} \right)^2 + \left(S^y_{n,i} \right)^2 \right] \end{aligned} \tag{4}$$

as given in Eq. 1 of the main text. All the main theoretical results presented in the main text are based on numerical minimizations of the energy stemming from this macrospin Hamiltonian.

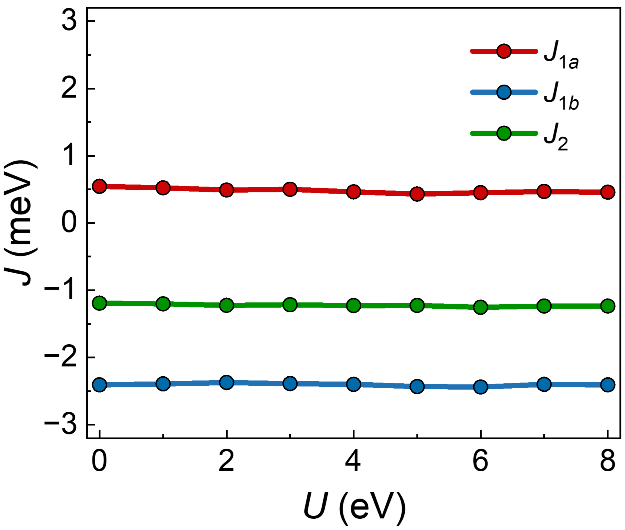

Supplementary Figure 1 | The calculated intralayer exchange interactions, J_{1a}, J_{1b} and J_2 as a function of chosen U.

Phenomenological derivation of THG_{CD} from a Ginzburg-Landau perspective

Without considering the magnetic structure, the $C_{2/m}$ point group of $CrPS_4$ leads to 4 non-zero components in the third order susceptibility tensor: $\chi^{(3)}$(xxxx), $\chi^{(3)}$(yyyy), $\chi^{(3)}$(xxyy) and $\chi^{(3)}$(yyxx). However, when taking the spin-spiral structure into consideration, the components of the third-order susceptibility tensor become independent in general, with likely all the 81 components non-vanishing. This makes it greatly challenging to disentangle the contributing tensor components in our THG_{CD} experiments. To phenomenologically derive a connection between the circular dichroism THG_{CD} and the spin chirality $\mathbf{j}$, we take a Ginzburg-Landau perspective and write out terms in the third order polarization which are linear in $\mathbf{j}$ and containing three instances of the electric field $\mathbf{E}(\omega)$. We also consider terms which include the $\mathbf{k}-$vector as could be obtained by including terms proportional to $\nabla \cdot \mathbf{E}$ or $\nabla \times \mathbf{E}$ in the polarization. The simplest terms we can consider are

$$\begin{aligned}\mathbf{P}^{(3)}(3\omega) =& \alpha\mathbf{E}\,(\mathbf{E}\cdot\mathbf{E}) + \beta\,(\mathbf{j}\times\mathbf{E})\,\mathbf{E}\cdot\mathbf{E} + \gamma\,(\mathbf{j}\times\mathbf{k})\times\mathbf{E}\,(\mathbf{E}\cdot\mathbf{E}) \\ &+ \eta\,(\mathbf{j}\cdot\mathbf{k})\,(\mathbf{E}\cdot\mathbf{E})\,\mathbf{E} + \xi\,(\mathbf{j}\times\mathbf{E})\cdot(\mathbf{k}\times\mathbf{E})\,\mathbf{E}.\end{aligned} \tag{5}$$

Let us first focus on the terms proportional to α and β. For a cicular polarization, we have $\mathbf{E}_{\pm} = (1, \pm i, 0)/\sqrt{2}$, and therefore $\mathbf{E}\cdot\mathbf{E} = 0$. Due to the transverse nature of the electric field, $\mathbf{k}\cdot\mathbf{E} = 0$, and hence, none of these terms could in principle survive and we would expect zero third order polarization $\mathbf{P}^{(3)}(\omega) = 0$. However, in doing so, we have not considered the in-plane C_{2v} symmetry of the magnetic model for $CrPS_4$. To do so in a simplistic manner, we consider that the in-plane anisotropy of $CrPS_4$ encodes a response which is described by a tensor $\mathbf{Q}$. Doing so, we can instead include terms of the form $\mathbf{P}^{(3)}(3\omega) = \alpha\mathbf{E}\,(\mathbf{E}\cdot\mathbf{Q}\cdot\mathbf{E}) + \beta\,(\mathbf{j}\times\mathbf{E})\,(\mathbf{E}\cdot\mathbf{Q}\cdot\mathbf{E})$. The intensity for σ^{+}-polarized light is therefore given by

$$\begin{aligned}I_{+}(3\omega) =& |\mathbf{P}_{+}^{(3)}(3\omega)|^2 \\ =& |\alpha|^2|C|^2|E|^2 + |\beta|^2(\mathbf{j}\times\mathbf{E})\cdot(\mathbf{j}\times\mathbf{E}^*)|C|^2 + 2i\Im\,(\alpha^*\beta)\,|C|^2\mathbf{E}^*\cdot(\mathbf{j}\times\mathbf{E}),\end{aligned} \tag{6}$$

where to simplify the notation we have introduced $C = \mathbf{E}\cdot\mathbf{Q}\cdot\mathbf{E}$. The intensity $I_{-}(3\omega)$ therefore reduces to

$$I_-(3\omega) = |\alpha|^2|C|^2|E|^2 + |\beta|^2(\mathbf{j}\times\mathbf{E})\cdot(\mathbf{j}\times\mathbf{E}^*)|C|^2 + 2i\Im\left(\alpha^*\beta\right)|C|^2\mathbf{E}\cdot(\mathbf{j}\times\mathbf{E}^*)\,. \tag{7}$$

Thus, we have a direct term $I^{\rightarrow} = |\alpha|^2|C|^2|E|^2 + |\beta|^2(\mathbf{j}\times\mathbf{E})\cdot(\mathbf{j}\times\mathbf{E}^*)|C|^2$, and a cross term $I^{\times} = 2\Im\left(\alpha^*\beta\right)|C|^2\mathbf{j}\cdot\mathbf{h}$ where $\mathbf{h} = i\left(\mathbf{E}^*\times\mathbf{E}\right)$ is called the light helicity. Now, the intensity difference stemming from this reduces to

$$I_+ - I_- = I^{\rightarrow} + I^{\times} - \left(I^{\rightarrow} - I^{\times}\right) = 2I^{\times} = 4\Im\left(\alpha^*\beta\right)|C|^2\mathbf{j}\cdot\mathbf{h}, \tag{8}$$

which tells us that the intensity difference becomes directly proportional to the coupling between chirality $\mathbf{j}$ and light helicity $\mathbf{h}$. Similarly, to calculate $I_+ + I_-$, we can first simplify $(\mathbf{j}\times\mathbf{E})\cdot(\mathbf{j}\times\mathbf{E}^*) = j^2|E|^2 - |\mathbf{j}\cdot\mathbf{E}|^2$, and thus we have

$$\begin{aligned} I_+ + I_- &= I^{\rightarrow} + I^{\times} + \left(I^{\rightarrow} - I^{\times}\right) = 2I^{\rightarrow} \\ &= 2|C|^2\left[|E|^2\left(|\alpha|^2 + |\beta|^2 j^2\right) - |\alpha|^2\,|\mathbf{j}\cdot\mathbf{E}|^2\right]. \end{aligned} \tag{9}$$

We therefore have

$$\mathrm{THG_{CD}} = \frac{I_+ - I_-}{I_+ + I_-} = \frac{4\Im\left(\alpha^*\beta\right)(\mathbf{j}\cdot\mathbf{h})}{2\left[\left(|\alpha|^2 + |\beta|^2 j^2\right)|E_0|^2 - |\beta|^2\,|\mathbf{j}\cdot\mathbf{E}|^2\right]} \tag{10}$$

Let us compute the even part of this. Note that $\mathbf{j}\cdot\mathbf{h} = \pm\mathbf{j}_z|E_0|^2$, and also that $|\mathbf{j}\cdot\mathbf{E}|^2 = |\mathbf{j}\cdot\frac{1}{\sqrt{2}}\left(1,\pm i,0\right)E_0|^2 = j_{\|}^2 E_0^2/2$. We have

$$\mathrm{THG_{CD}} = \frac{2\Im\left(\alpha^*\beta\right) j_z}{|\alpha|^2 + |\beta|^2\left(j_z^2 + \frac{1}{2}j_{\|}^2\right)}. \tag{11}$$

This means that the contribution will be odd in j_z. Let's now look at terms involving the propagation vector $\mathbf{k}$ which is proportional to the $\hat{\mathbf{z}}$ direction. The additional terms allowed by symmetry which include such a vector read

$$\mathbf{P}_k^{(3)}(3\omega) = C\left[\gamma\left(\mathbf{j}\times\mathbf{k}\right)\times\mathbf{E} + \eta\left(\mathbf{j}\cdot\mathbf{k}\right)\mathbf{E}\right] + \xi\left(\mathbf{j}\times\mathbf{E}\right)\cdot\left(\mathbf{k}\times\mathbf{E}\right)\mathbf{E}. \tag{12}$$

Simplifying $(\mathbf{j}\times\mathbf{E})\cdot(\mathbf{k}\times\mathbf{E}) = \mathbf{j}\cdot\mathbf{k}\,(\mathbf{E}\cdot\mathbf{E}) - (\mathbf{j}\cdot\mathbf{E})\,(\mathbf{E}\cdot\mathbf{k})$. This term vanishes for both σ^+ and σ^- polarizations. Furthermore, as before, since no direct terms contribute to $I^+ - I^-$, we can focus at first on the cross terms. In particular, simplifying further $(\mathbf{j}\times\mathbf{k})\times\mathbf{E} = \mathbf{k}\,(\mathbf{j}\cdot\mathbf{E}) - \mathbf{j}\,(\mathbf{k}\cdot\mathbf{E}) = \mathbf{k}(\mathbf{j}\cdot\mathbf{E})$, hence we are left with

$$\mathbf{P}_k^{(3)}(3\omega) = C\left[\left[\alpha + \eta k j_z\right]\mathbf{E} + \beta\,(\mathbf{j}\times\mathbf{E}) + \gamma\mathbf{k}\,(\mathbf{j}\cdot\mathbf{E})\right]. \tag{13}$$

As we saw, cross terms of $I^\times$ constituted from α and β contribute with a Lorentzian in j_z. We now have a similar situation with with $\alpha \longrightarrow \alpha + \eta k j_z$, yielding a contribution to the cross terms

$$I^\times_{(1)} = 4|C|^2\Im\left(\left[\alpha + \eta k j_z\right]^*\beta\right) j_z \tag{14}$$

Finally, we have to analyze the two cross terms for I_+, which read $\left[\alpha + \eta k j_z\right]\gamma^*\mathbf{E}\cdot\mathbf{k}\,(\mathbf{j}\cdot\mathbf{E}^*)$ and $\beta\gamma^*\,(\mathbf{j}\times\mathbf{E})\cdot\mathbf{k}\,(\mathbf{j}\cdot\mathbf{E}^*)$. Due to the transverse nature of the electric field $\mathbf{E}\cdot\mathbf{k} = 0$, the first of these terms vanishes. Then, we have only the term

$$I_+^{(2)} = |C|^2\beta\gamma^*\,(\mathbf{j}\times\mathbf{E})\cdot\mathbf{k}\,(\mathbf{j}\cdot\mathbf{E}^*) + |C|^2\gamma^*\beta\,(\mathbf{j}\times\mathbf{E}^*)\cdot\mathbf{k}\,(\mathbf{j}\cdot\mathbf{E}). \tag{15}$$

Using the circularly polarized definitions $(\mathbf{j}\times\mathbf{E})\cdot\mathbf{k}\,(\mathbf{j}\cdot\mathbf{E}^*) = -ikj_\parallel^2|E_0|^2/2$, we have

$$I^{+,\times}_{(2)} = C^2 k j_\parallel^2 |E_0|^2\Im\left(\beta\gamma^*\right). \tag{16}$$

Similarly, we have

$$\begin{aligned} I^{-,\times}_{(2)} =& |C|^2\beta\gamma^*\,(\mathbf{j}\times\mathbf{E}^*)\cdot\mathbf{k}\,(\mathbf{j}\cdot\mathbf{E}) + |C|^2\gamma^*\beta\,(\mathbf{j}\times\mathbf{E})\cdot\mathbf{k}\,(\mathbf{j}\cdot\mathbf{E}^*) \\ =& -|C|^2 k j_\parallel^2|E_0|^2\Im\left(\beta\gamma^*\right). \end{aligned} \tag{17}$$

This results in $I^\times_{(2)} = I^{+,\times}_{(2)} - I^{-,\times}_{(2)} = 2C^2kj_\parallel^2|E_0|^2\Im\left(\beta\gamma^*\right)$. As before, for the denominator, we have to compute the direct terms

$$I^{\rightarrow} = C^2|E_0|^2\left(|\alpha+\eta k j_z|^2+|\beta|^2 j_z^2+|\gamma|^2 k^2 j_{\|}^2\right). \tag{18}$$

And from cross terms, for which, again, we only have to calculate $\beta\gamma^*\left(\mathbf{j}\times\mathbf{E}\right)\cdot\mathbf{k}\left(\mathbf{j}\cdot\mathbf{E}^*\right)$ + h.c. but recalling that $\left(\mathbf{j}\times\mathbf{E}\right)\cdot\mathbf{k}\left(\mathbf{j}\cdot\mathbf{E}^*\right) = i j_{\|}^2|E_0|^2$, this contribution vanishes. This yields

$$\begin{aligned}\mathrm{THG_{CD}} =& \frac{I^+-I^-}{I^++I^-} = \frac{I^{\times}_{(1)}+I^{\times}_{(2)}}{I^{\rightarrow}}\\ =& \frac{2\Im\left(\alpha^*\beta\right) j_z+2\Im\left(\eta^*\beta\right) k j_z^2+\Im\left(\beta\gamma^*\right) k j_{\|}^2}{|\alpha+\eta k j_z|^2+|\beta|^2\left(j_z^2+\frac{1}{2}j_{\|}^2\right)+|\gamma|^2k^2 j_{\|}^2}.\end{aligned} \tag{19}$$

Thus, we find that we obtain both an even contribution proportional to $j_{\|}$ and an odd contribution proportional to j_z in the third harmonic dichroic response. It is worth noting that the frequency dependence present in this response is two-fold: Firstly, all the Landau-Ginzburg expansion coefficients may have some underlying frequency dependence; secondly, frequency can enter directly into the tensor via $\omega = kc$. This latter dependence is purely kinematical, and hence any additional resonant behavior may be explained only using an underlying microscopic description that could account for the potential frequency dependence of the expansion coefficients.

Description in spin spiral phases

AFM coplanar spiral. In the antiferromagnetic coplanar spiral state, spins in each layer are arranged antiferromagnetically and rotate gradually from layer to layer, forming a spiral with a well-defined propagation vector along the interlayer direction. All spins lie within a single plane, and there is no out-of-plane canting. From a side view, the spin directions rotate smoothly between adjacent layers, while from a top view the antiferromagnetic arrangement within each layer is preserved. This configuration supports a finite vector spin chirality due to the non-collinear interlayer rotation, despite the absence of a net magnetization.

AFM conical spiral. The AFM conical spiral evolves from the coplanar spiral upon the application of a magnetic field along the easy axis. In this phase, spins retain their antiferromagnetic character but acquire a uniform out-of-plane canting, forming a conical envelope as they rotate along the spiral direction. From a side view, the spins precess around the field direction with a

finite cone angle, while from a top view the in-plane spiral component remains visible. This state simultaneously hosts antiferromagnetic order, finite spin chirality, and a small net magnetization along the field direction.

FM canted spiral. At higher magnetic fields, the system transitions into a ferromagnetically canted spiral state. In this configuration, spins are predominantly aligned along the field direction, giving rise to a ferromagnetic component, while a residual spiral modulation persists due to competing interlayer exchange interactions. From a side view, spins are largely polarized, whereas from a top view, a weak non-collinear modulation remains. The spin chirality in this phase is reduced compared to the AFM spiral states but does not vanish abruptly, reflecting the continuous evolution of the magnetic structure.

Sample preparation and characterization

Flux zone-grown $CrPS_4$ crystals are purchased from 2D Semiconductor. $CrPS_4$ flakes are mechanically exfoliated by the dry transfer technique using a PDMS stamp to silicon chips with 285 nm thick SiO_2. A long working distance objective captures microscopic images of flakes on the stamp and substrate, while mechanical micro-stages ensure precise alignment. To facilitate sample location under high-magnification objectives in the cryostat, markers are fabricated on silicon chips. By using maskless lithography (MLA150, Heidelberg Instruments) and physical vapor deposition (Angstrom), a 4-inch silicon wafer with markers (5 nm Ti and 50 nm Au) is successfully fabricated. Then, the wafer is cut into 5 mm chips by using the dicing saw (Disco DADdy) after spin-coating a PMMA protection layer. Before transferring the flakes, the chips are washed sequentially with acetone, followed by isopropyl alcohol. The thickness of the samples is characterized using atomic force microscopy with a Dimension Icon system from Bruker. The measurements provide high-resolution topographical data, enabling precise determination of sample thickness. The Raman spectrum is measured by a WITEC alpha 300 RA+ system at room temperature. A 532 nm continuous wave laser is focused by a 100X CF Plan Nikon objective (NA = 0.95), and the reflected Raman signal is collected by the same objective and sent to a spectrometer.

Nonlinear optical experimental setups

For room-temperature wavelength-dependent third harmonic generation (THG) measurement, a laser source with a 2 kHz repetition rate from an amplified Ti:sapphire femtosecond laser system (Spectra-Physics Solstice Ace) is utilized. A 40× objective lens (Nikon, NA = 0.75) focuses the incident light and collects the generated signal. Detection is carried out using a spectrometer (Andor) equipped with a photomultiplier tube (Hamamatsu), which is connected to a lock-in amplifier (Stanford Research Systems).

Linear or circular polarization-dependent optical THG measurements at different temperatures are carried out using a custom-built confocal optical microscope, operating in reflection geometry (Figure 2). The sample is mounted into a closed-loop cryostat (attoDRY 2100) equipped with superconducting magnets (-9 to 9 T). The femtosecond laser at 1575 nm (FemtoFiber smart 780) is focused onto the sample by a low-temperature objective from Attocube (LT-APO/Telecom, NA = 0.8). A half-wave plate mounted on a motorized rotation stage (KPRM1E/M) is employed to rotate the linear polarization of the pump light. The reflected THG signal is collected by the same objective. The signal is further coupled into a multimode fiber to the Andor Shamrock 750 spectrograph equipped with an electron multiply charge-coupled device (EMCCD, Newton 970).

To measure reflection circular dichroism and chiral THG response by using the same setup (Figure 2), a quarter-wave plate placed after the dichroic mirror is employed to switch the chirality of the light. The chirality is determined by the polarimeter (PAX1000VIS/M, PAX1000IR2/M, Thorlabs). A spectrally-filtered supercontinuum laser source (SuperK Extreme, NKT Photonics) and a 1575 nm femtosecond laser are employed for reflection circular dichroism (RCD) measurements. By using the setup shown in Figure 2, the signals are detected using an Andor spectrometer equipped with visible EMCCD and a near-infrared camera (XEVA-17343), respectively.

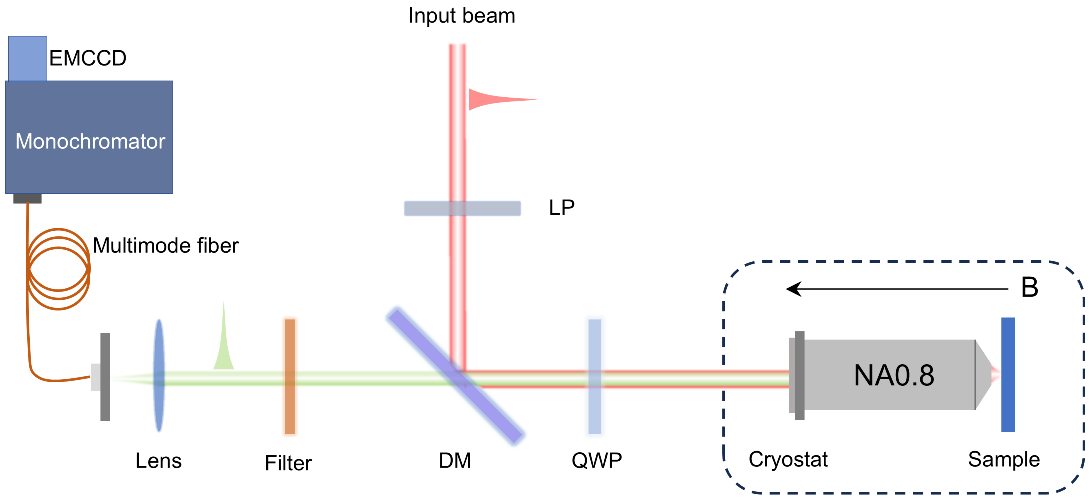

Supplementary Figure 2 | Schematic view of the experimental setup for THG measurement. DM: dichroic mirror; LP: linear polarizer; QWP: quarter wave plate; EMCCD: electron-multiplying charge-coupled devices; NA: numerical aperture

Supplementary Note 2

Atomic force microscope and Raman spectrum

The scanning area of atomic force microscopy is shown in Figure 3a , ensuring a detailed analysis of the surface morphology and uniformity. Figure 3a shows the section data from Figure 3a, where the thickness is determined to be ~ 12 nm.

Monolayer $CrPS_4$ consists of edge-sharing CrS_6 octahedra that create quasi-one-dimensional chains oriented along the *b*-axis, while these chains are linked along the *a*-axis via PS_4 tetrahedra (Figure 4). The dashed line exhibits straight edges that are preferentially aligned at an angle of 67.5° (112.5°), as observed in exfoliated $CrPS_4$ samples. We observe twelve Raman peaks in the range of 50 to 450 cm^{-1} as shown in Figure 5. Compared to the previous report [6], the observed Raman peaks closely match, with both peak positions and relative intensities aligning well.

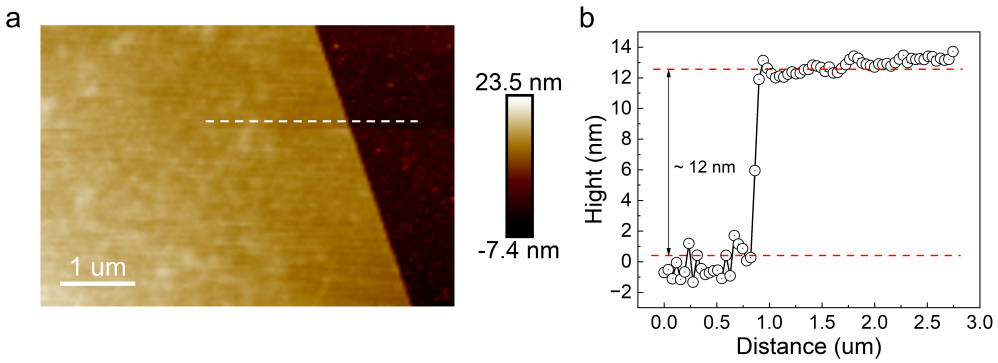

Supplementary Figure 3 | Thickness characterization of the 12 nm $CrPS_4$ flake. (a) Atomic force microscopy of as-measured $CrPS_4$ flake in the main text. (b) Extracted height data from (a).

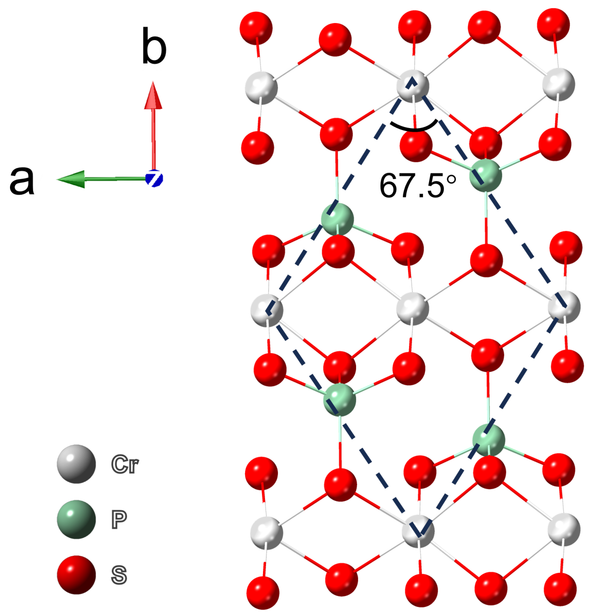

Supplementary Figure 4 | Crystal structure of monolayer $CrPS_4$.

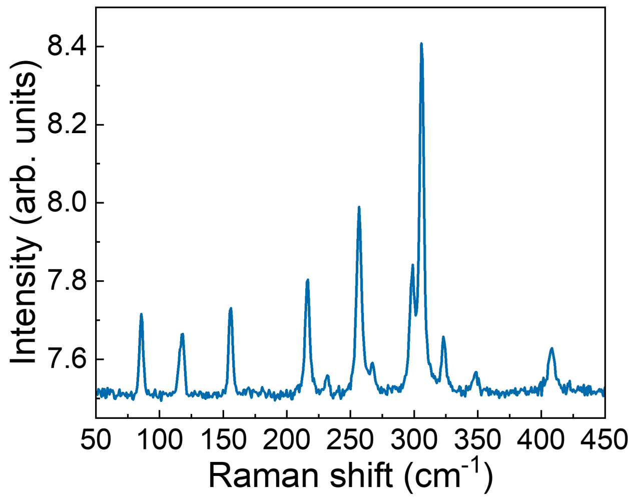

Supplementary Figure 5 | Raman spectrum of the 12 nm thick $CrPS_4$ flake at room tempearture.

THG excited by linearly polarized light

Figure 6 presents the wavelength-dependent THG response of exfoliated $CrPS_4$ under linearly polarized excitation along the b-axis at room temperature. The results show strong THG excited in the near-infrared range (1450–1600 nm). Figure 7 exhibits the linear polarization-dependent THG intensity measured over a temperature range of 2–300 K. The results reveal that the anisotropic THG response remains unchanged across different temperatures, indicating that the underlying atomic symmetry of $CrPS_4$ is preserved within this temperature range.

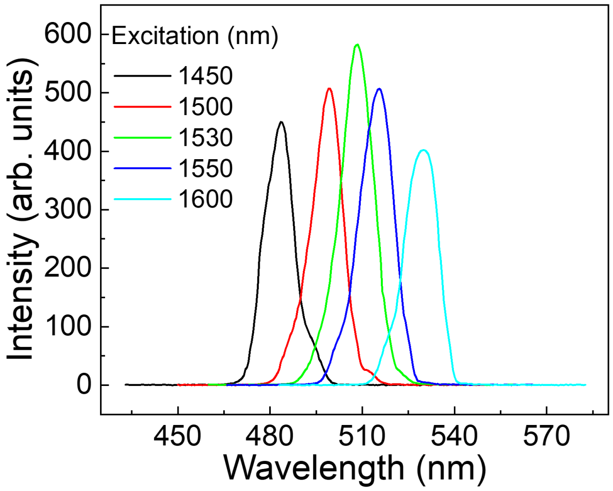

Supplementary Figure 6 | Wavelength-dependent THG from the 12 nm $CrPS_4$ flake excited by linearly polarized light at room temperature.

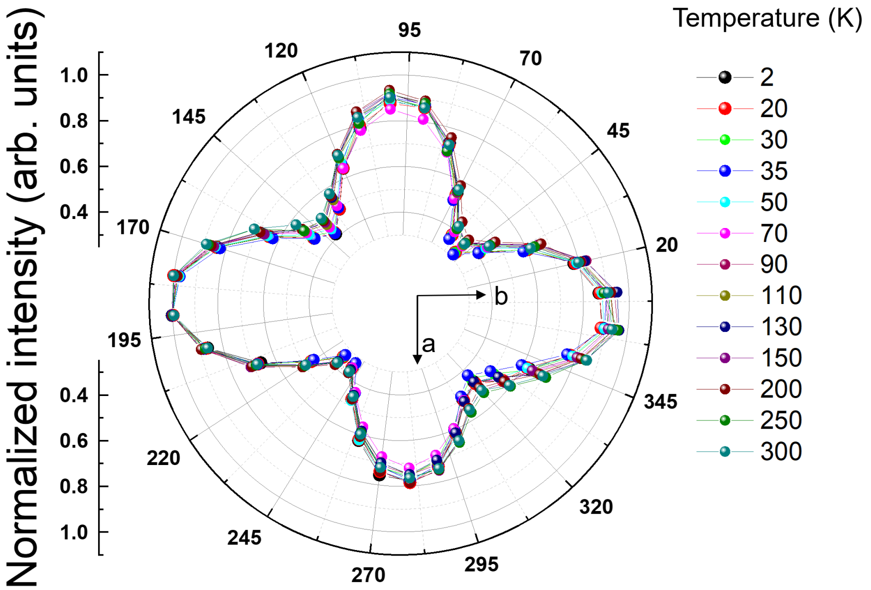

Supplementary Figure 7 | Linear polarization dependent THG intensity from the 12 nm $CrPS_4$ flake at a temperature range of 2-300 K. A half-wave plate is used to rotate the polarization of the fundamental wavelength.

Reflection circular dichroism and chiral THG response

The reflection circular dichroism (RCD), which measures the differential reflection of two beams with opposite chirality, was examined for the 12 nm thick $CrPS_4$ flake at a fundamental wavelength of 1575 nm using our system (Figure 10). The results show no chiral response, either with or without an applied magnetic field. We also measure the RCD spectrum in the range of 518–536 nm under an applied magnetic field (Figure 11a) and calculate the RCD relative to the applied magnetic field using the integrated intensity (Figure 11b). No chiral response is observed. These results indicate nonchiral atomic structures in $CrPS_4$.

The chiral THG response is measured by using the same cryostat with an outside optical setup displayed in Figure 2. Power-dependent THG circular dichroism (THG_{CD}) from a 12 nm thick flake is presented in Figure 13. The THG_{CD} is defined as $THG_{CD} = \frac{I^- - I^+}{I^- + I^+}$, where I^- represents the THG intensity excited by left-handed circular polarized (σ^-) light, and I^+ corresponds to the THG intensity generated by right-handed circular polarized (σ^+) light. The power-dependent measurements were conducted over a power range from ~4 to 35 mW (power density of 5.09-43.2 (GW/m^2), ensuring a comprehensive analysis of the nonlinear optical response. The THG_{CD} remains stable at approximately 16% across this range. The results indicate that the spin model in $CrPS_4$ is stable even with high excitation power.

Figure 14 presents THG imaging acquired using two piezo scanners from Attocube, which enable precise scanning of the sample position. These figures provide insight into the spatial distribution of THG signals and the circular dichroism (CD) contrast arising from different circular polarization states of the excitation light. For the two panels in Figure 14a, the color bar levels are the same. These panels display the spatially resolved THG response under excitation with left-handed circularly polarized (σ^-) and right-handed circularly polarized (σ^+) light. Comparing the two panels, the variations in THG intensity across the sample indicate differences in the chiroptical response. It is clear to see the edge between the flake and substrate because σ^+ beam excitation of the strong THG from $CrPS_4$. We can see stronger THG from σ^- excitation compared to that excited by σ^+ light. Figure 14b presents the calculated THG_{CD} based on the data from Figure 14a, which quantifies the difference in THG response between the two circularly polarized light excitations. The THG_{CD} map highlights regions with strong chiral optical activity, which is homogeneous across

the sample. The variations from the SiO_2/Si substrate region are because of the noise.

The steering and flipping process (Figure 15a) begins by exfoliating a $CrPS_4$ flake onto a polydimethylsiloxane (PDMS) stamp, which serves as a flexible support for the delicate flakes. The flake is then aligned and partially attached to the substrate by the micro-mechanical stage, allowing for controlled manipulation. Next, the PDMS stamp is lifted, steering the flake (normal) to the desired position on the target substrate. A new PDMS stamp, coated with polypropylene carbonate (PPC), is used to pick up the remaining portion of the flake from the first PDMS. Finally, the remaining flake (flipping) is transferred onto the same substrate. Figure 15b exhibits the opposite chirality behavior between the normal flake and flipped flake with and without a magnetic field.

Figure 16 exhibits the THG_{CD} measured across samples with varying thicknesses. Specifically, a thinner (~5 nm) flake was also measured and shows a reduced CD contrast. The THG_{CD} of the thinner (~5 nm) flake reaches only ~6% at 2 K, about half of the ~14% observed in the ~12 nm flake (Figure 16), and becomes negligible at room temperature (297 K), as shown in Figure 17. These results further highlight the important role of interlayer interactions in determining the THG_{CD} response. Figure 20 displays the extracted odd components of THG_{CD} as a function of B field and temperatures. The results further confirm the chiral THG behavior of the flake.

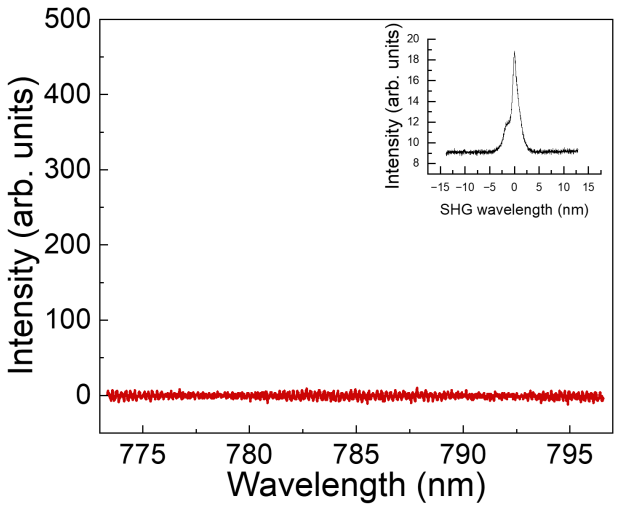

Supplementary Figure 8 | Spectrum in the second-harmonic generation range. The inset displays the second-harmonic generation (SHG) response of monolayer WS_2 as a reference.

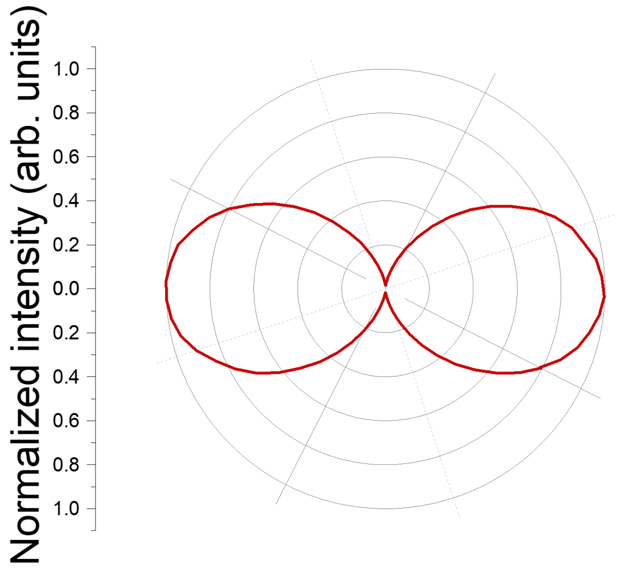

Supplementary Figure 9 | Output polarization analysis of the third-harmonic signal when excited by circularly polarized light.

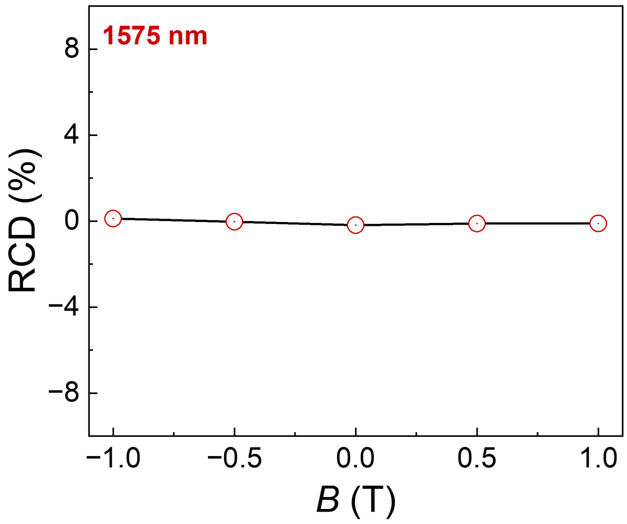

Supplementary Figure 10 | RCD at the fundamental wavelength of 1575 nm from a 12 nm $CrPS_4$ flake at temperature of 2 K. The results indicate that the crystal exhibits no measurable circular birefringence at 1575 nm.

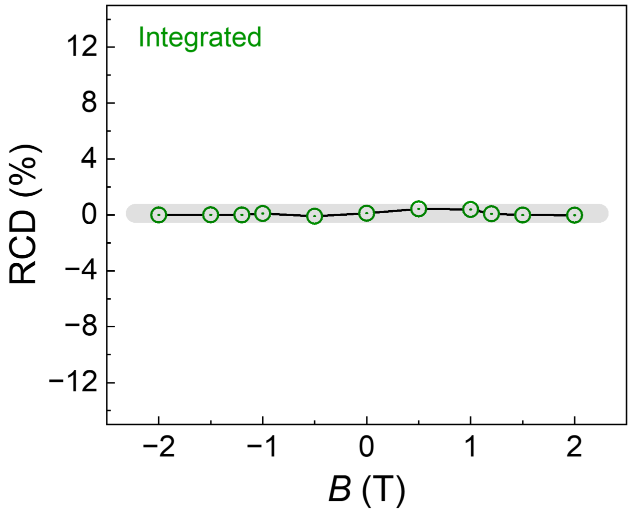

Supplementary Figure 11 | RCD by using integrated intensity at 525 nm under different magnetic fields. The results indicate that the crystal exhibits no measurable circular birefringence at 525 nm. Gray shading highlights the trend to guide the eye.

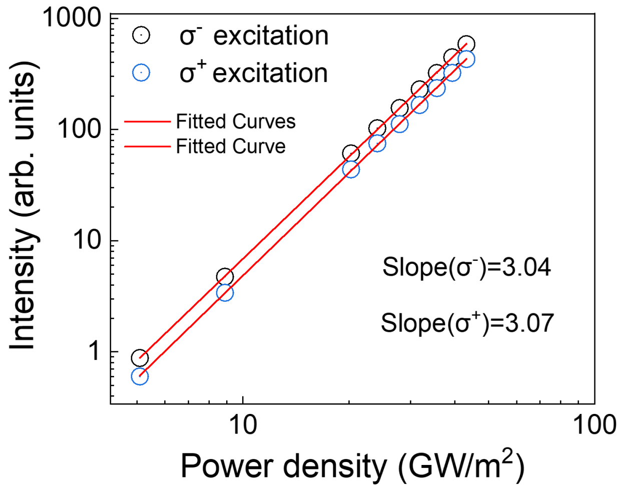

Supplementary Figure 12 | Power dependence of THG intensity from $CrPS_4$ under left- and right-circularly polarized excitation.

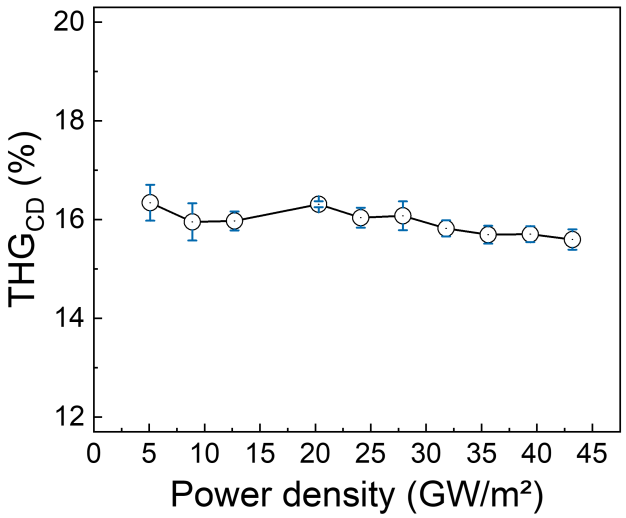

Supplementary Figure 13 | Power dependent THG_{CD} from the 12 nm thick $CrPS_4$ flake at temperature of 2 K. Error bars denote the standard error of the mean, calculated from five independent measurements.

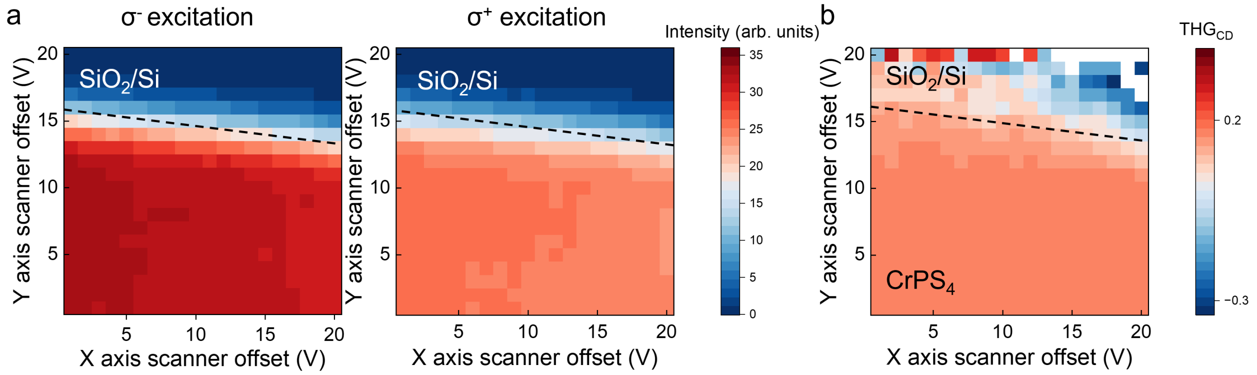

Supplementary Figure 14 | THG imaging of the 12 nm thick $CrPS_4$ flake at the temperature of 2 K. (a) Spatial mapping of THG intensity excited by left-handed circular polarized (σ^-) light and right-handed circular polarized (σ^+). (b) Calculated THG_{CD} based on the data from (a).

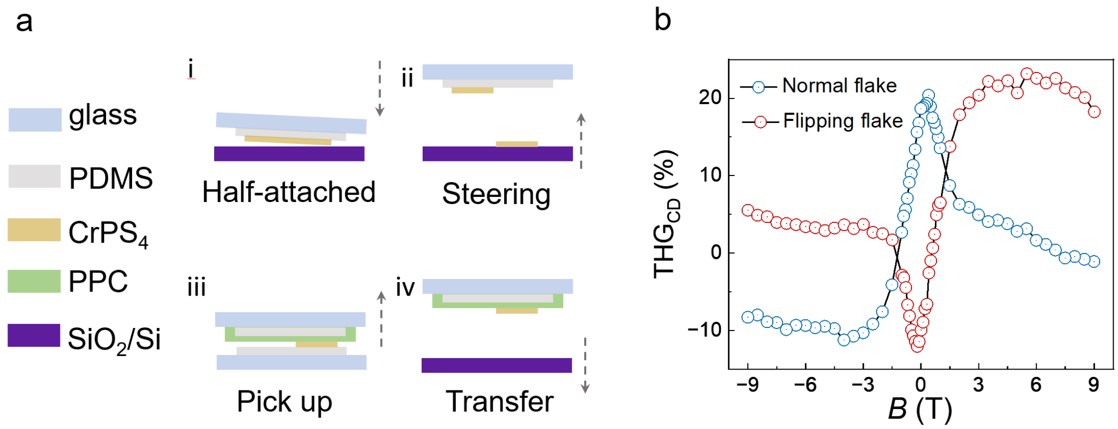

Supplementary Figure 15 | Steering and flipping process with magnetic-dependent THG_{CD}. (a) Schematic view of steering and flipping process at room temperature. (b) Magnetic-dependent THG_{CD} of the normal flake and flipping flake on the same substrate at the temperature of 2 K.

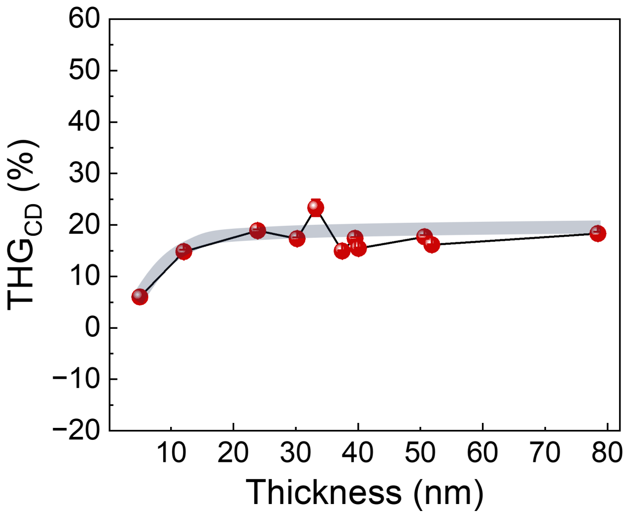

Supplementary Figure 16 | THG_{CD} from $CrPS_4$ with different thicknesses at the temperature of 2 K. Error bars denote the standard error of the mean, calculated from five independent measurements. Gray shading highlights the trend to guide the eye.

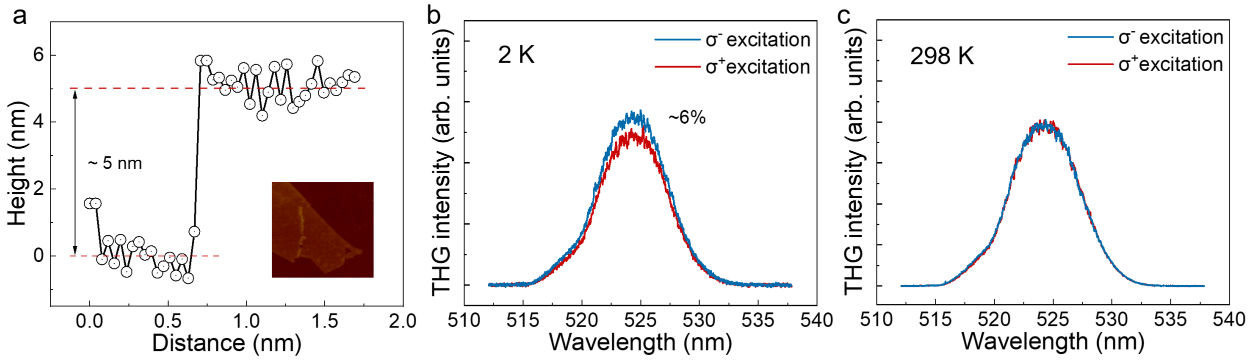

Supplementary Figure 17 | Thickness characterization and THG of a 5 nm $CrPS_4$ flake. (a) Height data from atomic force microscopy. The inset shows the image. THG spectra obtained from a 5 nm thin flake when excited by left- and right-circularly polarized light at (b) 2 K and (c) 298 K.

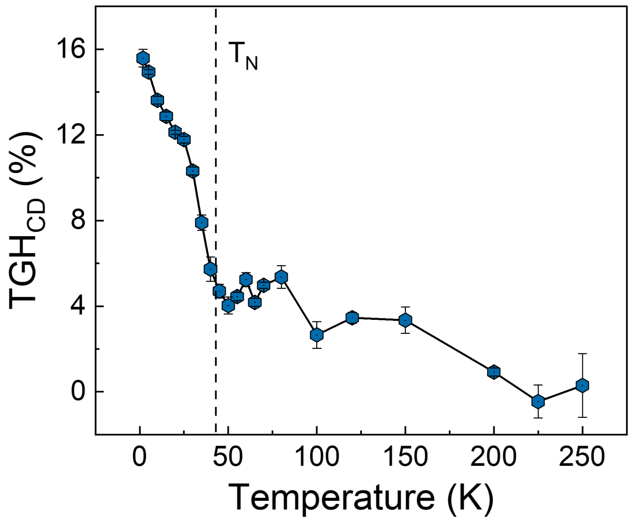

Supplementary Figure 18 | THG$_{CD}$ from a 12 nm thick $CrPS_4$ flake measured in the temperature range 2–250 K. T$_N$: Néel temperature. Error bars denote the standard error of the mean, calculated from five independent measurements.

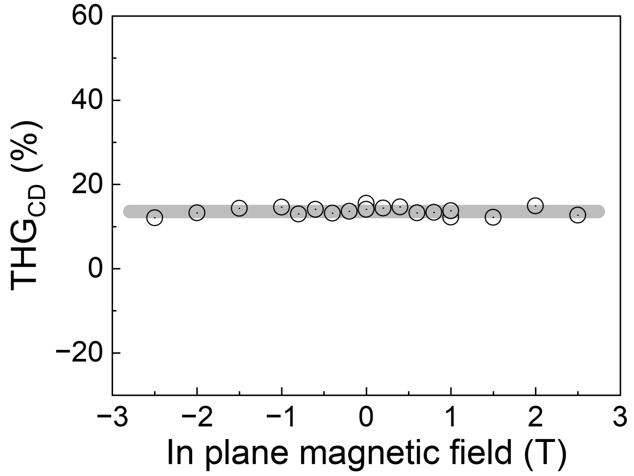

Supplementary Figure 19 | THG$_{CD}$ when applying in plane magnetic field of ±2.5 T. Gray shading highlights the trend to guide the eye.

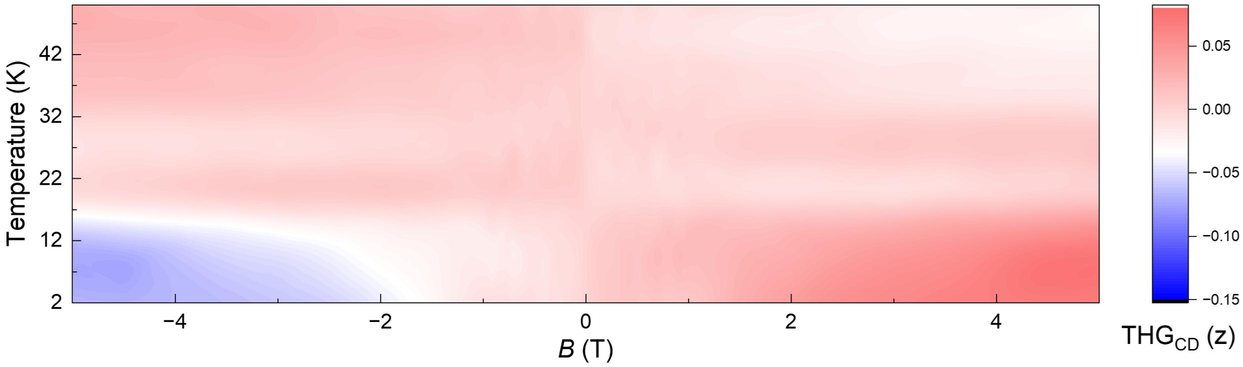

Supplementary Figure 20 | Mapping plot of odd components of THG_{CD} from the 12 nm $CrPS_4$ flake as a function of *B* field and temperature.

To the best of our knowledge, previous reports that magneto-optical Kerr effect (MOKE), reflective magnetic circular dichroism (RMCD), magnetic force microscopy (MFM), second harmonic generation (SHG), and neutron scattering techniques haven't discussed the correlation between the finite vector spin chirality and their signals. In our work, we emphasize that our conclusions are based on a combined analysis of THG_{CD} measurements and phenomenological modeling in spin chirality, and we regard the proposed chiral spiral state as a physically consistent interpretation.

Supplementary Table 1 | Comparison of different magnetic measurement methods

Method	Sensitive Quantity	Spatial Resolution	Typical Signal	Detected Structure	References
MOKE	Net magnetization	μm	μrad	FM domains, magnetic bubbles	[7]
RMCD	Net magnetization	μm	$< 1\%$	FM domains, magnetic bubbles	[8]
MFM	Magnetic stray field	nm	None	FM domains, magnetic bubbles	[9]
SHG	Magnetic order	μm	None	AFM domains, magnetic polygraphs	[10]
THG_{CD}	Finite vector spin chirality	μm	$> 10\%$	AFM coplanar spin spirals	This work

References

1. Peng, Y. *et al.* Magnetic structure and metamagnetic transitions in the van der Waals antiferromagnet $CrPS_4$. *Adv. Mater.* **32,** 202001200 (2020).
2. *The Elk code* `https://elk.sourceforge.io/`. (accessed 2024-17-01).
3. Soriano, D., Cardoso, C. & Fernández-Rossier, J. Interplay between interlayer exchange and stacking in CrI_3 bilayers. *Solid State Commun.* **299,** 113662 (2019).
4. Pakdel, S., Olsen, T. & Thygesen, K. S. Effect of Hubbard U-corrections on the electronic and magnetic properties of 2D materials: a high-throughput study. *npj Comput. Mater.* **11,** 18 (2025).
5. Menichetti, G., Calandra, M. & Polini, M. Electronic structure and magnetic properties of few-layer $Cr_2Ge_2Te_6$: the key role of nonlocal electron–electron interaction effects. *2D Mater.* **6,** 045042 (2019).
6. Kim, S., Lee, J., Lee, C. & Ryu, S. Polarized Raman spectra and complex Raman tensors of antiferromagnetic semiconductor $CrPS_4$. *J. Phys. Chem. C* **125,** 2691–2698 (2021).
7. Son, J. *et al.* Air-stable and layer-dependent ferromagnetism in atomically thin van der Waals $CrPS_4$. *ACS Nano* **15,** 16904–16912 (2021).
8. Meijer, M. J. *et al.* Chiral spin spirals at the surface of the van der Waals ferromagnet Fe_3GeTe_2. *Nano Letters* **20,** 8563–8568 (2020).
9. Cai, M. *et al.* Topological magneto-optical effect from skyrmions in two-dimensional ferromagnets. *ACS Nano* **18,** 20055–20064 (2024).
10. Sun, Z. *et al.* Resolving and routing magnetic polymorphs in a 2D layered antiferromagnet. *Nature Materials* **24,** 226–233 (2025).
