# Supplementary material for: Observation of tunable chiral spin textures with nonlinear optics
Source: Nat Commun. 2026 Jun 15;17:7576. doi: 10.1038/s41467-026-74328-x (PMC13415826; doi:10.1038/s41467-026-74328-x)
Supplement: Supplementary file 2 — Peer Review file [file 41467_2026_74328_MOESM2_ESM.pdf]

nature portfolio

Peer Review File

Observation of tunable chiral spin textures with nonlinear optics

Corresponding Author: Professor Zhipei Sun

This file contains all reviewer reports in order by version, followed by all author rebuttals in order by version.

Version 0:

Reviewer comments:

Reviewer #1

(Remarks to the Author)
In the manuscript titled “Observation of tunable chiral spin textures with nonlinear optics” by Huang et al., the authors report the observation of tunable chiral magnetic textures in van der Waals CrPS4 with nonlinear optics. First, the authors use theoretical simulations to demonstrate the existence of spin chirality in multilayer CrPS4. Furthermore, the authors measure the third-harmonic-generation (THG) signal in CrPS4 at the magnetic ordered state and show that the existence of spin chirality can induce a THG signal which can be tuned by an external magnetic field. The authors claim that this finding highlignt the potency of THG for probing non-collinear magnetic orders.

This manuscript reveals some interesting experimental observations, and the authors give some explanations. However, the generality of this effect and the generality of their explanation are not well- addressed. My detailed comments and questions are below.

1. How does the spin chirality and THG effect rely on layer numbers? What is the magnetic structure in few-layer CrPS4, e.g. three layers, four layers?

2. When describing the structure and magnetic configuration of CrPS4, the authors need to mention the direction of magnetic easy axis.

3. Are there any connections between the quasi-one-dimensional chains, spin chirality, and the observed THG?

4. Regarding the three magnetic configurations (AFM coplanar spiral, AFM conical spiral, and FM canted spiral), more descriptions or figures from different perspectives need to be added.

5. The meaning of A_// in Eq(1) is not explained.

6. The authors used J_// and J_perp to represent the spin chirality. Considering the large in-plane anisotropy in CrPS4, what does the vector form of J look like?

7. The authors measured the THG signal under normal incident. Which component matters to the THG_CD? Is the above results works for every component of THG susceptibility? What is the role of each component in THG susceptibility?

8. If the spin configuration develops chirality, the spin configuration already breaks the inversion. This has also been mentioned in line 140-142. Why SHG is not observed and reported?

9. What is the meaning of Eq(2)? What is the meaning of all the parameters, and how general is the equation? Could this equation be applied to another canted spin structure?

10. Different from the static spin configuration, optical responses such as THG is a spectrum of frequency. The THG experiments are done at 1575 nm. What is the optical feature at this wavelength? How does the observed THG_CD vary with frequency? If the response is very sensitive to frequency, it would be meaningless to use a Landau model based on the static spin structure to describe it optical response.

11. In Fig 2e, why the figure is not symmetric w.r.t. B field?

12. How to understand the sensitivity of THG w.r.t. in-plane and out-of-plane B field?

13. As the authors show in Fig 4b-c, the behavior of THG_CD can be decomposed into a B-field-even term and a B-field-odd term. Is the reversing of B field equivalent to a time reversing operation? Can the THG in general be composed of a time-reversal-even term and a time-reversal-odd term?

14. Fig 4d. What is the physical meaning of the derivative of THG_CD? Why the derivative is a odd function in B while the THG_CD is not?

Reviewer #2

(Remarks to the Author)
The manuscript reports the observation of circular dichroism in third-harmonic generation (THG-CD) in $CrPS_4$ and attributes it to a chiral non-collinear magnetic ground state driven by competing interlayer exchange interactions. The use of nonlinear optical spectroscopy to probe magnetic chirality is potentially significant.
However, several fundamental conceptual and experimental issues remain unresolved. In its current form, the manuscript overstates the exclusivity of the THG-CD interpretation, relies on the centrosymmetric crystal structure arguments, and does not sufficiently reconcile its claims with existing experimental literature on $CrPS_4$, particularly regarding inversion symmetry, layer-number dependence, and previously reported optical responses. Addressing these concerns is essential before the conclusions can be substantiated. Hence, I cannot recommend this paper for acceptance with this version. The list of my main comments and questions is given below.

1. The manuscript assumes that $CrPS_4$ is centrosymmetric and further claims that no second-harmonic generation (SHG) signal is detected, using this as key evidence for structural inversion symmetry. This assumption is then used to exclude structural contributions to the observed THG-CD.
However, multiple previous studies have reported broken inversion symmetry in $CrPS_4$ at both the structural and magnetic levels, accompanied by clear SHG signals. This inconsistency is not a minor detail but directly impacts the central interpretation of the THG-CD signal. In non-centrosymmetric systems, structural chirality and magneto–structural mixing terms are symmetry-allowed and may all contribute to THG-CD.
Therefore, if inversion symmetry is already broken, THG-CD cannot be uniquely attributed to magnetic chirality without additional constraints, which are currently not sufficiently addressed in the manuscript.
2. Prior studies on $CrPS_4$ have demonstrated a pronounced odd–even layer number dependence, as demonstrated by techniques including MOKE and SHG, implying thickness-dependent magnetic symmetry and layer-sensitive magnetic order.
In contrast, the present work assumes a single chiral spiral magnetic ground state persisting over a broad thickness range, which would not be expected to have odd–even layer number dependence. The manuscript does not explain why the magnetic response probed by THG differs from that observed in previous studies. This inconsistency requires further clarification and discussion.
3. While the manuscript proposes a non-collinear spiral magnetic ground state, the supporting evidence relies almost entirely on THG-CD measurements and phenomenological modeling. Reported in van der Waals magnets, non-collinear magnetic structures have been directly imaged or resolved using techniques such as MOKE, RMCD, and MFM microscopy, which provide spatially resolved information on magnetic domains.
The manuscript suggests that THG-CD reveals the magnetic ground state of $CrPS_4$. However, THG-CD is not a direct magnetic structure determination technique. At present, the claim of a revised magnetic ground state—especially one differing from magnetic structures previously resolved by neutron scattering—remains indirect. If a new magnetic ground state is proposed, more direct experimental evidence for non-collinear magnetism is required.
4. According to the manuscript, a chiral spiral magnetic structure emerges at low temperature. This would generally be expected to lower the symmetry and potentially break inversion symmetry compared to the high-temperature phase.
In this case, a question arises as to whether this symmetry change is reflected in SHG measurements. Whether SHG-CD is enhanced or newly allowed in the low-temperature chiral phase compared to room temperature?
5. In the temperature-dependent measurements, the THG-CD signal does not vanish completely above the Néel temperature. What is the physical origin of the non-zero THG-CD signal observed above the Néel temperature?

Version 1:

Reviewer comments:

Reviewer #1

(Remarks to the Author)
I am satisfied with most of the responses except the following two comments:

In response R1-8, the authors claim no SHG measured in the spin-canting structure. From symmetry aspect, canted spin order that breaks PT symmetry can generate SHG in electric-dipole approximation. This effect has been predicted (Sci. Adv. 11, eadu6562) and experimentally observed (arXiv:2509.13542) in CrSBr. It is possible that due to the weak SOC, this effect

is difficult to observe in SHG. I think the authors should make this point clearer. In addition to THG, there are other transport and optical measurements that are sensitive to spin chirality, which I think are closely related to this work and should be cited (Phys. Rev. Lett. 124, 096602, Commun. Phys. 4, 99, Phys. Rev. Lett. 130, 166702, Sci. Adv. 11, eadu6562).

In response R 1-10, the authors says “The fundamental wavelength used in our experiments (1575 nm, corresponding to a photon energy of around 0.79 eV) lies well below the dominant interband optical transitions of CrPS4, placing the measurement in a non-resonant regime.” As in THG, 0.79 eV light can induce resonance even at 3*0.79 eV, I think this is already in the resonant regime.

Reviewer #2

(Remarks to the Author)
The authors have provided a detailed response and revised the manuscript, which has improved the clarity of their arguments, particularly regarding the magnetic origin of the THG-CD signal and its distinction from SHG and MOKE probes. However, one critical issue raised in my initial review remains insufficiently addressed, and a new contradiction has emerged from their own response.
1. The authors argue that the observed THG-CD originates from a chiral spin texture driven by competing interlayer exchange interactions—a multilayer magnetic effect. If this were an intrinsic bulk magnetic property, one would expect a clear dependence of the THG-CD signal amplitude on the number of magnetic layers (thickness), especially when transitioning from a few layers to the multilayer regime. However, the Figure R10 show "within our experimentally accessible thickness range, the THG-CD response exhibits consistent behavior". This lack of thickness sensitivity is paradoxical for a phenomenon claimed to arise from interlayer couplings.
2. While the authors correctly note that techniques like MFM are insensitive to antiferromagnetic textures, the claim of a "non-collinear chiral ground state" still relies entirely on a single optical probe (THG-CD) and phenomenological modeling. The lack of direct structural evidence remains a significant weakness for a paper claiming the observation of a new magnetic texture.
I cannot recommend publication in the current state and I hope the author will adequately address the above issues in the next revision.

Version 2:

Reviewer comments:

Reviewer #2

(Remarks to the Author)
The authors have responded carefully to the issues I raised. Specifically, they have made descriptive corrections regarding the thickness-dependent data. In addition to the THG measurements, the authors also performed SHG measurements on CrPS4, but no SHG signal was detected. However, both theoretical and experimental studies indicate that symmetry breaking (either lattice or magnetic) in CrPS4 can give rise to large SHG signals (e.g., Adv. Optical Mater. 2024, 2400943). In these related studies, a pronounced thickness dependence was also observed, which aligns well with the authors' analysis of the origins of the SHG and THG signals. I recommend that the authors further compare this part of their work with the existing literature to ensure an accurate and appropriate description.

Open Access This Peer Review File is licensed under a Creative Commons Attribution 4.0 International License, which permits use, sharing, adaptation, distribution and reproduction in any medium or format, as long as you give appropriate credit to the original author(s) and the source, provide a link to the Creative Commons license, and indicate if changes were made.
In cases where reviewers are anonymous, credit should be given to 'Anonymous Referee' and the source.
The images or other third party material in this Peer Review File are included in the article's Creative Commons license, unless indicated otherwise in a credit line to the material. If material is not included in the article's Creative Commons license and your intended use is not permitted by statutory regulation or exceeds the permitted use, you will need to obtain permission directly from the copyright holder.
To view a copy of this license, visit https://creativecommons.org/licenses/by/4.0/

Response letter

Dear Editor and Reviewers,

We sincerely thank the editor and all reviewers for their valuable suggestions and constructive comments. Their feedback has been very helpful in improving the quality of our manuscript. Both reviewers find our work of interest. Indeed, Reviewer #1 mentioned that "*This manuscript reveals some interesting experimental observations...*". Reviewer #2 mentioned that "*The use of nonlinear optical spectroscopy to probe magnetic chirality is potentially significant...*".

We are pleased to respond to their comments line by line and have revised our manuscript based on the responses below. We are also grateful to the reviewers for their constructive comments and the time and effort they dedicated to evaluating our work. In this response letter, we provide a detailed point-by-point reply to all the reviewers. Reviewer comments are marked in black and italic, our responses are presented in blue, and the corresponding quotes or revisions from the manuscript and supplementary information are highlighted in red.

Reviewer #1 (Comments for the Author):

Comments from Reviewer 1-0:

In the manuscript titled "Observation of tunable chiral spin textures with nonlinear optics" by Huang et al., the authors report the observation of tunable chiral magnetic textures in van der Waals $CrPS_4$ with nonlinear optics. First, the authors use theoretical simulations to demonstrate the existence of spin chirality in multilayer $CrPS_4$. Furthermore, the authors measure the third-harmonic-generation (THG) signal in $CrPS_4$ at the magnetic ordered state and show that the existence of spin chirality can induce a THG signal which can be tuned by an external magnetic field. The authors claim that this finding highlights the potency of THG for probing non-collinear magnetic orders.

This manuscript reveals some interesting experimental observations, and the authors give some explanations. However, the generality of this effect and the generality of their explanation are not well- addressed. My detailed comments and questions are below.

Response R1-0:

Many thanks to the reviewer for the valuable evaluation of our manuscript. We are very grateful to the reviewer for the kind recognition of our work, and we sincerely appreciate the reviewer's insightful suggestions, which have been invaluable in improving the manuscript.

Comments from Reviewer 1-1:

1. How does the spin chirality and THG effect rely on layer numbers? What is the magnetic structure in few-layer $CrPS_4$, e.g. three layers, four layers?

Response R1-1:

We thank the reviewer for this question. In the present work, our study focuses on $CrPS_4$ samples within the thickness range investigated experimentally, where the interlayer exchange interactions are well developed. In this regime, the chiral spin correlations probed by THG_{CD} originate from competing interlayer spin interactions and therefore reflect a multilayer magnetic behavior rather than layer-parity effects.

Importantly, within our experimental thickness range, the THG_{CD} signal shows a consistent behavior in Fig. R1 of this reply letter (Figure 16 of the Supplementary information), supporting the physical picture that the observed chiral magnetic correlations arise from interlayer interactions rather than from finite-size or odd–even layer-number effects. Actually, we have spent a lot of time exfoliating the few-layer $CrPS_4$ using different exfoliation methods. The thinnest flake that we can obtain is ~5 nm. Fig. R2 of this reply letter (Figure 17 in the Supplementary information) shows the thickness characterization and THG of a ~5 nm $CrPS_4$ flake.

Manuscript revision:

Line 164-168, page 10: "Within our experimentally accessible thickness range, the THG_{CD} response exhibits consistent behavior with no clear layer-parity dependence. Unlike MOKE and SHG, which are sensitive to net magnetization or layer-parity–dependent symmetry breaking. THG is even allowed in centrosymmetric systems. It suggests that the observed chiral signal is governed primarily by competing near and next-near interlayer exchange interactions that persist in multilayer $CrPS_4$."

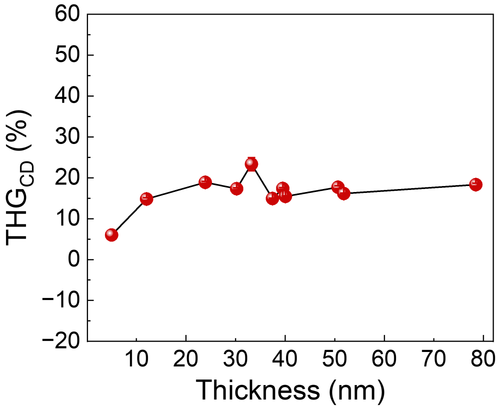

Fig. R1 (Figure 16 of the supplementary information). *THG$_{CD}$ from $CrPS_4$ with different thicknesses at the temperature of ~2 K.*

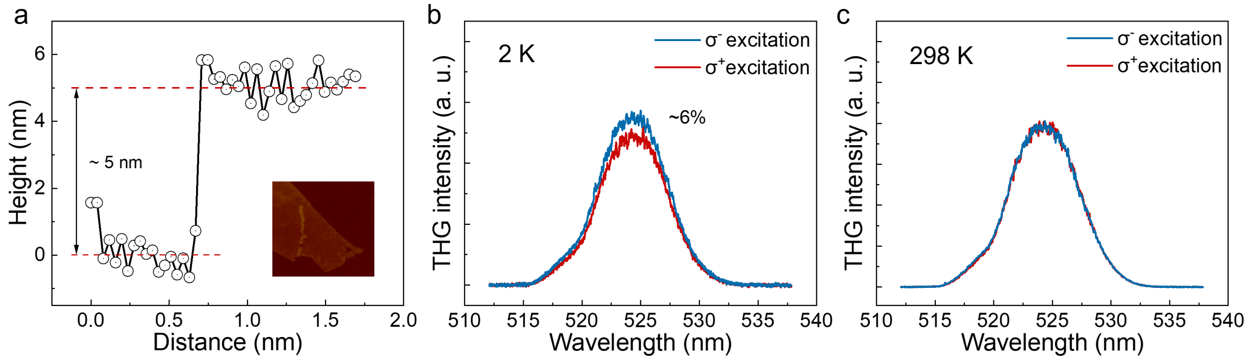

Fig. R2 (Figure 17 of the supplementary information). *Thickness characterization and THG of a ~5 nm $CrPS_4$ flake. (a) Height data from atomic force microscopy. The inset shows the image. THG Spectra obtained from a ~5 nm thin flake when excited by left- and right-circularly polarized light at (b) ~2 K and (c) ~298 K.*

Comments from Reviewer 1-2:

2. When describing the structure and magnetic configuration of $CrPS_4$, the authors need to mention the direction of magnetic easy axis.

Response R1-2:

We thank the reviewer for pointing this out. We have revised the manuscript to explicitly state the direction of the magnetic easy axis of $CrPS_4$. Based on previous experimental studies, the magnetic easy axis of $CrPS_4$ lies along the crystallographic c axis (out-of-plane direction). This information has now been included in the section describing the crystal structure and magnetic configuration to improve clarity and completeness.

Manuscript revision:

Line 54-56, page 3: "$CrPS_4$ is an air-stable vdW magnet known for its A-type antiferromagnetic (AFM) behavior, where ferromagnetic (FM) layers couple antiferromagnetically along the c-axis, corresponding to the magnetic easy axis."

Comments from Reviewer 1-3:

3. Are there any connections between the quasi-one-dimensional chains, spin chirality, and the observed THG?

Response R1-3:

We thank the reviewer for this insightful question. The quasi-one-dimensional (quasi-1D) chain structure of $CrPS_4$ plays an indirect but important role in the emergence of spin chirality and the observed THG response.

Structurally, the quasi-1D Cr–S chains define the dominant intralayer exchange pathways, which stabilize robust ferromagnetic order within each vdW layer. As shown by our DFT calculations, these intralayer interactions do not introduce magnetic frustration and therefore do not themselves generate spin chirality. Instead, they justify a coarse-grained description in which each layer can be treated as a single macro-spin.

The spin chirality in the manuscript in $CrPS_4$ arises from competing interlayer exchange interactions between these ferromagnetically ordered layers. Specifically, the competition between nearest-neighbor ($J_{\perp 1}$) and next-nearest-neighbor ($J_{\perp 2}$) antiferromagnetic interlayer couplings stabilizes a non-collinear spin-spiral state along the out-of-plane direction. The resulting spin texture carries a finite vector spin chirality associated with the relative rotation between adjacent layers.

The observed THG_{CD} signal is sensitive to this interlayer chiral spin texture, rather than to the quasi-1D chains themselves. As shown in Figure 4 from the main text, the observed THG_{CD} is highly correlated with the vector spin chirality. The role of the quasi-1D structure is to enforce strong intralayer collinearity within each layer.

Manuscript revision:

Line 64-67, page 3: "These chains are interconnected along the a-axis through PS_4 tetrahedra. Structurally, the quasi-one-dimensional chains define the dominant intralayer exchange pathways, which stabilize robust ferromagnetic order within each vdW layer. The magnetic moments of $CrPS_4$ within each layer are placed in a rectangular lattice formed by the Cr^{3+} ions."

Comments from Reviewer 1-4:

4. Regarding the three magnetic configurations (AFM coplanar spiral, AFM conical spiral, and FM canted spiral), more descriptions or figures from different perspectives need to be added.

Response R1-4:

We thank the reviewer for this helpful suggestion. We have expanded the textual descriptions of the three magnetic configurations (AFM coplanar spiral, AFM conical spiral, and FM canted spiral) to improve clarity. We have added these to the Supplementary information.

Manuscript revision:

Line 103-104, pages 5-6: "...a chiral magnetic texture driven by the competition between $J_{\perp 1}$ and $J_{\perp 2}$. This corresponds to a coplanar spin spiral phase (see the Supplementary information for more descriptions). Beyond the spin-flop critical field (B_{c1})..."

Supplementary information revision:

Line 138-159, pages 9-10: "AFM coplanar spiral. In the antiferromagnetic coplanar spiral state,

spins in each layer are arranged antiferromagnetically and rotate gradually from layer to layer, forming a spiral with a well-defined propagation vector along the interlayer direction. All spins lie within a single plane, and there is no out-of-plane canting. From a side view, the spin directions rotate smoothly between adjacent layers, while from a top view the antiferromagnetic arrangement within each layer is preserved. This configuration supports a finite vector spin chirality due to the non-collinear interlayer rotation, despite the absence of a net magnetization.

AFM conical spiral. The AFM conical spiral evolves from the coplanar spiral upon the application of a magnetic field along the easy axis. In this phase, spins retain their antiferromagnetic character but acquire a uniform out-of-plane canting, forming a conical envelope as they rotate along the spiral direction. From a side view, the spins precess around the field direction with a finite cone angle, while from a top view the in-plane spiral component remains visible. This state simultaneously hosts antiferromagnetic order, finite spin chirality, and a small net magnetization along the field direction.

FM canted spiral. At higher magnetic fields, the system transitions into a ferromagnetically canted spiral state. In this configuration, spins are predominantly aligned along the field direction, giving rise to a ferromagnetic component, while a residual spiral modulation persists due to competing interlayer exchange interactions. From a side view, spins are largely polarized, whereas from a top view, a weak non-collinear modulation remains. The spin chirality in this phase is reduced compared to the AFM spiral states but does not vanish abruptly, reflecting the continuous evolution of the magnetic structure."

Comments from Reviewer 1-5:

5. The meaning of $A_{||}$ in Eq(1) is not explained.

Response R1-5:

We thank the reviewer for pointing this out. In Eq. (1), $A_{||}$ denotes the *in-plane magnetic anisotropy*, which penalizes spin components lying in the plane and thus favors alignment along the out-of-plane easy axis of each $CrPS_4$ layer. It represents the energy cost for tilting spins away from the easy-axis orientation within the plane and is included in the spin Hamiltonian to account for the

weak in-plane anisotropy. We have added this definition explicitly in the revised manuscript to clarify the meaning of $A_{\|}$. .

Manuscript revision:

Line 82-84, page 5: "$A_{\|}$ denotes the in-plane magnetic anisotropy. The nearest ($J_{\perp 1}$) and next-nearest ($J_{\perp 2}$) interlayer exchange interactions in $CrPS_4$ ..."

Comments from Reviewer 1-6:

6. The authors used $J_{\|}$ and $J_{\perp}$ to represent the spin chirality. Considering the large in-plane anisotropy in $CrPS_4$, what does the vector form of J look like?

Response R1-6:

The vector $\boldsymbol{J} = (J_x, J_y, J_z)$ will have components which vary in magnitude according to the external applied magnetic field. For zero magnetic field, the presence of a coplanar spiral implies that $J_z = 0$. The J_x and J_y components are not fully specified, but rather only the in-plane magnitude $J_{\|} = \sqrt{J_x^2 + J_y^2}$ is. This is because the structural anisotropy does not appear to be strong enough to induce any in-plane frustration. This means that there occurs a spontaneous symmetry breaking phenomena, where the ground state picks out a particular direction for the fully in-plane $\boldsymbol{J}$ vector at zero field. When the magnetic field is increased, the components $J_{\|}$ and J_z vary as depicted in Fig. 1 of the main text. In practice, this means that we mainly assume a vector $\boldsymbol{J} = (J_{\|}, 0, J_z)$ with an appropriate choice of xy axes in the macrospin model.

Comments from Reviewer 1-7:

7. The authors measured the THG signal under normal incident. Which component matters to the THG_{CD}? Is the above results works for every component of THG susceptibility? What is the role of each component in THG susceptibility?

Response R1-7:

We thank the reviewer for this question. In our experiments, the THG signal was measured under normal incidence with circularly polarized light. While we do not explicitly resolve the individual components of the third-order susceptibility tensor $\chi^{(3)}$, the measured THG_{CD} reflects a combination of the symmetry-allowed components that couple to the circular polarization and are sensitive to magnetic chirality.

The observed field and temperature-dependent behavior indicate that the THG_{CD} is dominated by magnetically sensitive contributions, even if the precise tensor components are difficult to individually identify. Therefore, the THG_{CD} provides a robust probe of the chiral spin correlations in $CrPS_4$ under the chosen experimental geometry.

Without considering the magnetic structure, the $C_{2/m}$ point group of $CrPS_4$ leads to 4 non-zero components in the third order susceptibility tensor: $\chi^{(3)}$(xxxx), $\chi^{(3)}$(yyyy), $\chi^{(3)}$(xxyy) and $\chi^{(3)}$(yyxx). However, when taking the spin-spiral structure into consideration, the components of the third-order susceptibility tensor become independent in general, with likely all the 81 components non-vanishing. This makes it greatly challenging to disentangle the contributing tensor components in our THG_{CD} experiments, which deserve further study. We have added to the Supplementary information.

Supplementary information revision:

Line 84-89, page 6. "Without considering the magnetic structure, the $C_{2/m}$ point group of $CrPS_4$ leads to 4 non-zero components in the third order susceptibility tensor: $\chi^{(3)}$(xxxx), $\chi^{(3)}$(yyyy), $\chi^{(3)}$(xxyy) and $\chi^{(3)}$(yyxx). However, when taking the spin-spiral structure into consideration, the components of the third-order susceptibility tensor become independent in general, with likely all the 81 components non-vanishing. This makes it greatly challenging to disentangle the contributing tensor components in our THG_{CD} experiments."

Comments from Reviewer 1-8:

8. If the spin configuration develops chirality, the spin configuration already breaks the inversion. This has also been mentioned in line 140-142. Why SHG is not observed and reported?

Response R1-8:

We thank the reviewer for this insightful question. While a chiral spin configuration indeed breaks inversion symmetry at the level of the magnetic order, this does not necessarily imply the appearance of a detectable SHG signal. Electric-dipole SHG is governed primarily by the symmetry of the crystal lattice, and in bulk $CrPS_4$ the underlying lattice remains centrosymmetric (space group $C_{2/m}$) [Acta Crystallogr. Sect. B 33, 285 (1977)]. As a result, electric-dipole SHG can remain exceptionally weak even though there is a small breaking of inversion symmetry at the electronic level.

Magnetically induced SHG can, in principle, arise through higher-order processes (such as magnetic-dipole or electric-quadrupole contributions). However, these contributions are typically several orders of magnitude weaker than electric-dipole SHG and fall below our experimental detection limit. While we can observe clear THG from $CrSP_4$ and SHG from monolayer WS_2, no measurable SHG signal was observed in our experiments at 2 K shown in Fig. R3 (Figure 8 of the Supplementary information) from the Supplementary information.

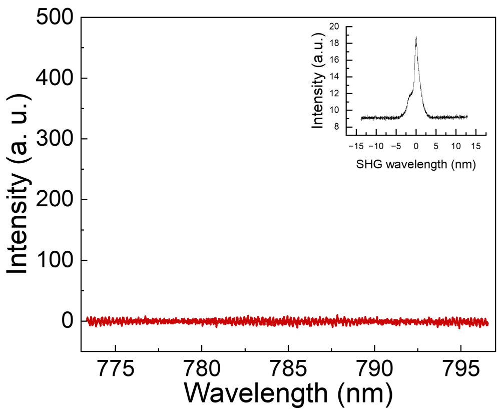

Fig. R3 (Updated Figure 8 of the supplementary information). *Spectrum in the second-harmonic generation range. The inset displays the second-harmonic generation (SHG) response of monolayer* WS_2 *as a reference.*

Comments from Reviewer 1-9:

9. What is the meaning of Eq(2)? What is the meaning of all the parameters, and how general is the equation? Could this equation be applied to another canted spin structure?

Response R1-9:

We thank the referee for this question. Eq. (2) serves as a phenomenological description of the origin of the THG_{CD} formula, and it is obtained using a Landau-Ginzburg prescription detailed in the supplemental information. In essence, all possible terms allowed by symmetry in the third-order power $\boldsymbol{P}^{(3)}(\omega)$ are written down with expansion coefficients whose value is phenomenologically adjusted, and the most general possible expression for the THG_{CD} is obtained. The parameters $\alpha, \beta, \gamma, \eta$ in Eq. (2) correspond precisely to phenomenological Landau-Ginzburg expansion coefficients that multiply terms allowed by symmetry in the third-order polarisation $\boldsymbol{P}^{(3)}(\omega)$. This expression is obtained for C_2 in-plane symmetric systems and for the third order. Systems with different structural symmetry have to be handled with care so as to preserve only terms allowed by symmetry in the expansion, as detailed in the Supplementary information. Slightly more detail is now added to the main text after the equation in order to clarify both the meaning of the expansion coefficients and the symmetry necessary for the applications of Eq. (2).

Manuscript revision:

Line 205-210, page 11: "where α, β, γ and η are Landau-Ginzburg expansion coefficients and k stands in for the magnitude of the wave-vector. In particular, α precedes the simplest possible third order term $\boldsymbol{E}(\boldsymbol{E} \cdot \boldsymbol{E})$, β precedes a symmetry allowed term $\boldsymbol{j} \times \boldsymbol{E}$ while γ and η precede terms that include $\boldsymbol{j} \cdot \boldsymbol{k}$ and $\boldsymbol{j} \times \boldsymbol{k}$ respectively. Despite the slightly more complex form of this expression, it is clear that the odd part of the THG_{CD} as a function of magnetic field will be dominated by j_z whereas the even part is dominated by $j_{\|}$, which justifies our choice of order parameters for the magnetic transitions, as detailed in the following discussion."

Comments from Reviewer 1-10:

10. Different from the static spin configuration, optical responses such as THG is a spectrum of frequency. The THG experiments are done at 1575 nm. What is the optical feature at this wavelength? How does the observed THG_{CD} vary with frequency? If the response is very sensitive to frequency, it would be meaningless to use a Landau model based on the static spin structure to describe its optical response.

Response R1-10:

We thank the reviewer for raising this important point regarding the frequency dependence of the THG response. The fundamental wavelength used in our experiments (1575 nm, corresponding to a photon energy of around 0.79 eV) lies well below the dominant interband optical transitions of $CrPS_4$, placing the measurement in a non-resonant regime. In this regime, the THG response is primarily governed by virtual electronic transitions and symmetry-allowed nonlinear susceptibilities, rather than by sharp resonant optical features. An experimental realization of a full frequency-dependent THG spectroscopy would indeed provide additional insight into the nature of this response. At present, however, the lack of tunable laser sources at other wavelengths for the cryostat in our lab currently constrains the study and motivates future experimental developments. Our Landau-Ginzburg analysis is intended to phenomenologically establish symmetry-allowed coupling between spin chirality and the THG_{CD}. Using our approach, the magnitude of the wave-vector can provide limited information about eventual frequency dependence via $\omega = kc$. However, it is worth noting that the expansion coefficients utilized in this expansion may also be frequency dependent when the symmetry-determined coupling structure remains valid. Thus, as pointed out by the referee, resonance effects would necessarily have to be accounted for using more detailed microscopic models for THG, which motivate future study. To address the reviewer's comments, we have added the following to the supplementary information.

Supplementary information revision:

Line 132-137, page 9: "It is worth noting that the frequency dependence present in this response is two-fold: Firstly, all the Landau-Ginzburg expansion coefficients may have some underlying frequency dependence; secondly, frequency can enter directly into the tensor via $\omega = kc$. This latter dependence is purely kinematical, and hence any additional resonant behavior may be explained only using an underlying microscopic description that could account for the potential frequency dependence of the expansion coefficients."

Comments from Reviewer 1-11:

11. In Fig 2e, why the figure is not symmetric w.r.t. B field?

Response R1-11:

We thank the reviewer for this question. The asymmetry of the THG_{CD} curve with respect to the applied magnetic field arises from the interplay of contributions associated with the in-plane and out-of-plane vector spin chirality, as illustrated in Fig. R4 (Figure 4 a-c of the main text). The in-plane vector spin chirality exhibits an even dependence on the magnetic field, whereas the out-of-plane component shows an odd dependence (Fig. R4 b and c). The superposition of these two contributions therefore leads to the observed asymmetric THG_{CD} (Fig. R4 a). And the THG_{CD} is defined as the normalized difference between the THG intensities excited by left- and right-handed circularly polarized light. Fig. R5 (Figure 2e of the main text) shows the THG intensity as a function of the applied magnetic field, which underlies the field-dependent asymmetry observed in the THG_{CD}.

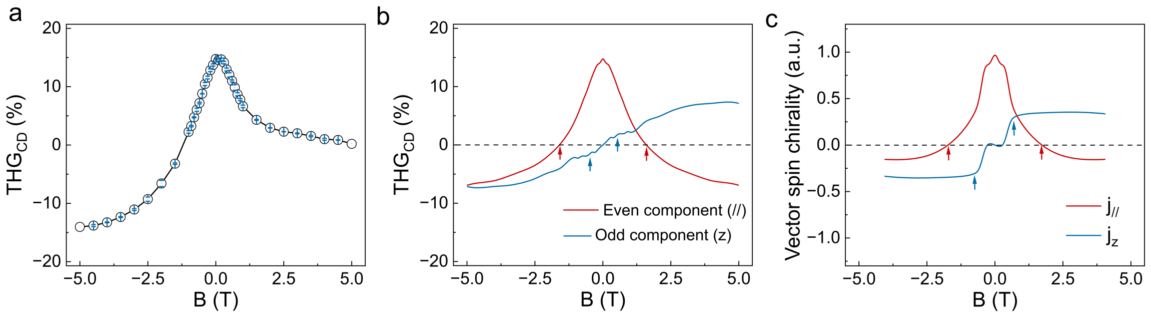

Fig. R4 (Figure 4 a-c of the main text). *Correlation between chiral THG measurement with the spin-feather structures in $CrPS_4$.* (a) Experimental results of THG_{CD} for the ~ 12 nm thick sample vs. B field at 2 K. (b) Decomposition of the THG_{CD} into even and odd components. Blue arrows mark changes in the behavior of the odd component, consistent with the predicted spin-flop transition at field B_{c1}, while red arrows mark sign switches in the even component of THG_{CD} at critical field B_{c2}. (c) Predicted in-plane ($j_{||}$) and out-of-plane (j_z) vector spin chirality averaged over an 18-layer (i.e., ~ 12 nm) flake. A clear analogy is verified between the odd and even components of the THG_{CD} and the vector spin chiralities, which serve here as a measure of the flake's chirality and non-collinearity.

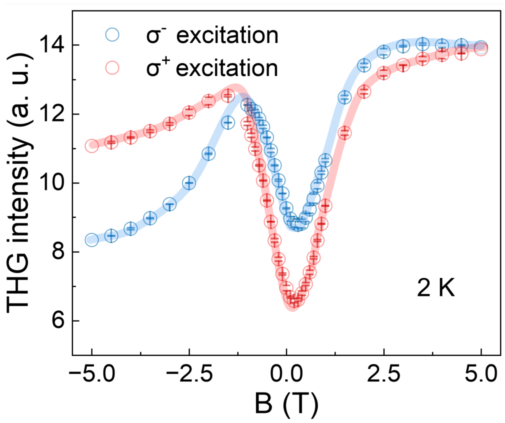

Fig. R5 (Figure 2e of the main text) *Out-of-plane magnetic field dependence of the chiral THG intensity at the temperature of ~ 2 K.*

Comments from Reviewer 1-12:

12. How to understand the sensitivity of THG w.r.t. in-plane and out-of-plane B field?

Response R1-12:

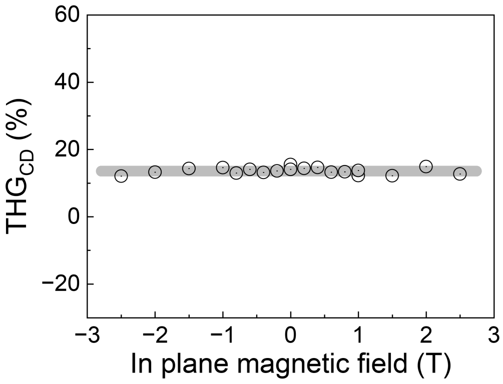

Fig. R6 (Figure 19 in the Supplementary information). *THG_{CD} when applying in plane magnetic field of ±2.5 T.*

We thank the reviewer for this question. $CrPS_4$ is an out-of-plane antiferromagnet, with the magnetic easy axis along the crystallographic c-axis. Consequently, the THG_{CD} is primarily sensitive to magnetic fields applied along this out-of-plane direction, which perturb the interlayer spin arrangement and modify the net spin chirality.

In contrast, in-plane fields have less effect on the antiferromagnetic spin structure within each layer due to the hard axis. Experimentally, within the accessible in-plane field range (up to 2.5 T), no significant change in THG_{CD} is observed in Fig. R6 (Figure 19 from Supplementary information), consistent with the spin configuration and symmetry of the $CrPS_4$ system.

Comments from Reviewer 1-13:

13. As the authors show in Fig 4b-c, the behavior of THG_{CD} can be decomposed into a B-field-even term and a B-field-odd term. Is the reversing of B field equivalent to a time reversing operation? Can the THG in general be composed of a time-reversal-even term and a time-reversal-odd term?

Response R1-13:

We thank the reviewer for raising this important conceptual point. Reversing the external magnetic field B is not equivalent to a time-reversal operation. Time reversal reverses all spin moments and magnetic order parameters, whereas magnetic-field reversal only changes the direction of the applied field and does not, in general, enforce a full time-reversed magnetic configuration.

We emphasize that we do not claim that the THG process itself can be uniquely separated into independent time-reversal-even and time-reversal-odd optical responses. Rather, the experimentally observed THG_{CD} can be phenomenologically decomposed into components that are even or odd with respect to magnetic-field reversal. This decomposition reflects how different magnetic order parameters respond to the applied field, rather than a strict symmetry classification of the nonlinear susceptibility under time reversal.

In particular, in $CrPS_4$, distinct components of the spin chirality (in-plane and out-of-plane) evolve differently under magnetic-field reversal due to magnetic anisotropy and competing interlayer interactions. Their combined contributions lead to THG_{CD} signals with different field parities. The observed B-even and B-odd behaviors therefore arise from the field-dependent evolution of the magnetic configuration, not from a direct correspondence between field reversal and time reversal.

Comments from Reviewer 1-14:

14. Fig 4d. What is the physical meaning of the derivative of THG_{CD}*? Why the derivative is a odd function in B while the* THG_{CD} *is not?*

Response R1-14:

We thank the reviewer for this question. Applying a derivative typically helps reveal fine features and weak transitions. Here, taking the derivative of $\mathrm{THG_{CD}}$ with respect to magnetic field highlights the field sensitivity of the chiral magnetic response, quantifying how rapidly $\mathrm{THG_{CD}}$ varies as the magnetic configuration evolves under the applied field. In particular, taking the derivative emphasizes the field range where the spin configuration undergoes the most pronounced reorientation or canting, such as across a spin-flop or crossover region.

The $\mathrm{THG_{CD}}$ itself contains both even-in-field and odd-in-field contributions, arising from the coexistence of in-plane vector spin chirality terms as well as out of plane spin chirality. As a result, the raw $\mathrm{THG_{CD}}$ curve is generally asymmetric but not strictly odd in the magnetic field.

Reviewer #2 (Comments for the Author):

Comments from Reviewer 2-0:

The manuscript reports the observation of circular dichroism in third-harmonic generation (THG_{CD}) in $CrPS_4$ and attributes it to a chiral non-collinear magnetic ground state driven by competing interlayer exchange interactions. The use of nonlinear optical spectroscopy to probe magnetic chirality is potentially significant. However, several fundamental conceptual and experimental issues remain unresolved. In its current form, the manuscript overstates the exclusivity of the THG_{CD} interpretation, relies on the centrosymmetric crystal structure arguments, and does not sufficiently reconcile its claims with existing experimental literature on $CrPS_4$, particularly regarding inversion symmetry, layer-number dependence, and previously reported optical responses. Addressing these concerns is essential before the conclusions can be substantiated. Hence, I cannot recommend this paper for acceptance with this version. The list of my main comments and questions is given below.

Response R2-0:

We thank the reviewer for their careful reading of the manuscript and for their constructive and detailed comments. We appreciate the recognition of the potential significance of using nonlinear optical spectroscopy to probe magnetic chirality in $CrPS_4$.

Comments from Reviewer 2-1:

1. The manuscript assumes that $CrPS_4$ is centrosymmetric and further claims that no second-harmonic generation (SHG) signal is detected, using this as key evidence for structural inversion symmetry. This assumption is then used to exclude structural contributions to the observed THG_{CD}. However, multiple previous studies have reported broken inversion symmetry in $CrPS_4$ at both the structural and magnetic levels, accompanied by clear SHG signals. This inconsistency is not a minor detail but directly impacts the central interpretation of the THG_{CD} signal. In non-centrosymmetric systems, structural chirality and magneto–structural mixing terms are symmetry-allowed and may all contribute to THG_{CD}. Therefore, if inversion symmetry is already broken, THG_{CD} cannot be uniquely attributed to magnetic chirality without additional constraints, which are currently not

sufficiently addressed in the manuscript.

Response R2-1:

We thank the reviewer for raising this important concern regarding inversion symmetry and its implications for the interpretation of the THG_{CD} signal. The material crystallizes in a monoclinic structure, with a space group $C_{2/m}$ [Acta Crystallogr. Sect. B 33, 285 (1977)] or C2 [Acta Crystallogr. Sect. B 33 1399 (1977)]. As discussed in the reference [PRB 103, 224407 (2021)] and as far as we know, there is no clear conclusion about the ambiguity of the crystal structure.

We acknowledge that previous studies have reported SHG signals in $CrPS_4$, which may originate from extrinsic effects such as surface or interface symmetry breaking, strain, defects, or higher-order (magnetic-dipole or electric-quadrupole) SHG processes. Magnetically induced SHG can, in principle, arise through higher-order processes (such as magnetic-dipole or electric-quadrupole contributions). However, these contributions are typically several orders of magnitude weaker than electric-dipole SHG and fall below our experimental detection limit. While we can observe clear THG, no measurable SHG signal was observed in our experiments at 2 K shown in Fig. R7 (updated Figure 8 from the Supplementary information). And the crystal does not possess any circular birefringence shown from reflection circular dichroism (RCD) in Figs. R8 and R9 (Figures 10 and 11 from the Supplementary information) and the discussion in reference [ACS Nano 15, 16904–16912 (2021)].

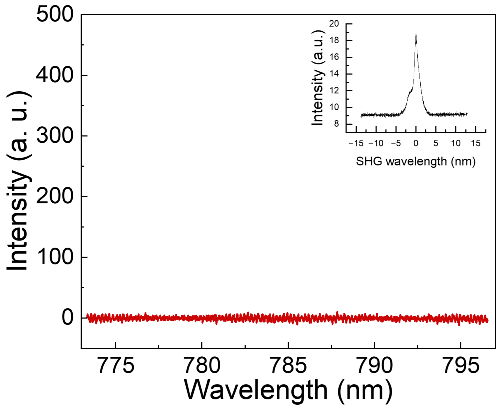

Fig. R7 (Updated Figure 8 of the supplementary information). *Spectrum in the second-harmonic generation range. The inset figure shows the second-harmonics generation from monolayer* WS_2.

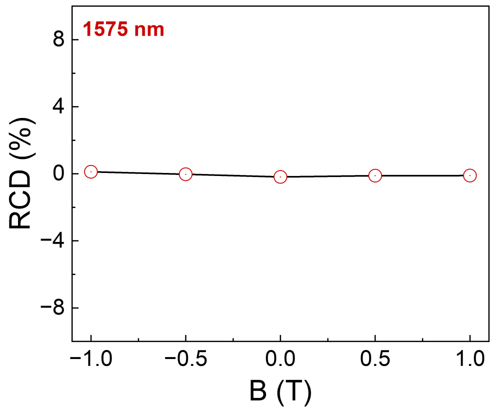

Fig. R8 (Figure 10 of the supplementary information). *RCD at the fundamental wavelength of 1575 nm from a 12 nm $CrPS_4$ flake at temperature of 2 K. The results indicate that the crystal exhibits no measurable circular birefringence at 1575 nm.*

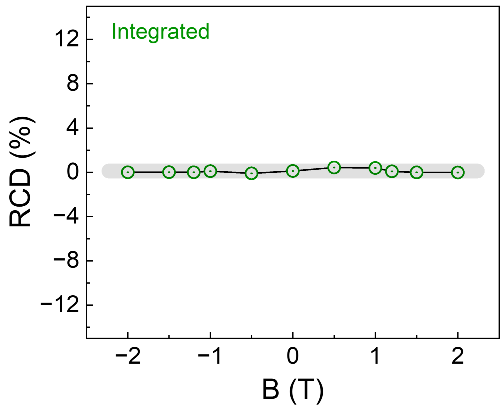

Fig. R9 (Figure 11 of the supplementary information). *RCD by using integrated intensity at 525 nm under different magnetic fields. The results indicate that the crystal exhibits no measurable circular birefringence at 525 nm.*

Importantly, our interpretation of THG_{CD} does not rely solely on the assumption of perfect inversion symmetry. Even if weak crystal structural inversion symmetry breaking were present, the associated structural chirality contributions would be expected to persist over a broad temperature range and to show weak temperature dependence. In contrast, the temperature dependence of the THG_{CD} provides strong evidence for its magnetic origin. The THG_{CD} signal closely follows the evolution of magnetic order, showing a pronounced enhancement in the magnetically ordered phase and a rapid suppression upon approaching and exceeding the Néel temperature. At sufficiently high temperatures, the THG_{CD} signal vanishes entirely.

This clear correlation between THG_{CD} and magnetic order indicates that the observed THG_{CD} is dominated by magnetic contributions, rather than by structural chirality. We have revised the manuscript to clarify this distinction and to more explicitly state the constraints under which THG_{CD} is interpreted as a probe of chiral magnetic correlations.

Manuscript revision:

Line 117-119, page 6. "It is noteworthy that no measurable second-harmonic generation is observed (Supplementary Fig. 8) within our system sensitivity limit, indicating a possible centrosymmetric crystal structure ($C_{2/m}$) for $CrPS_4$, consistent with a previous report [ref]."

Line 172-177, page 10. "We emphasize that the interpretation of the chiral THG signal does not depend on perfect inversion symmetry of the crystal structure. While even if weak structural inversion symmetry breaking cannot be completely excluded, any resulting structural chirality would be expected to exhibit weak temperature dependence. Instead, the THG_{CD} signal shows a pronounced enhancement in the magnetically ordered phase and vanishes well at higher temperatures. This behavior supports a dominant magnetic origin of the THG_{CD} response."

Comments from Reviewer 2-2:

2. Prior studies on $CrPS_4$ have demonstrated a pronounced odd–even layer number dependence, as demonstrated by techniques including MOKE and SHG, implying thickness-dependent magnetic symmetry and layer-sensitive magnetic order. In contrast, the present work assumes a single chiral spiral magnetic ground state persisting over a broad thickness range, which would not be expected to have odd–even layer number dependence. The manuscript does not explain why the magnetic

response probed by THG differs from that observed in previous studies. This inconsistency requires further clarification and discussion.

Response R2-2:

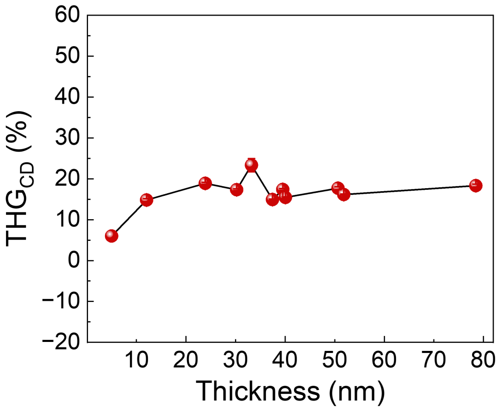

Fig. R10 (Figure 16 of the supplementary information). *THG_{CD} from $CrPS_4$ with different thicknesses at the temperature of ∼2 K.*

We thank the reviewer for raising this important point. Indeed, previous studies on $CrPS_4$ have reported a pronounced odd–even layer-number dependence using probes such as MOKE and SHG, which are highly sensitive to net magnetization and inversion symmetry, respectively. These techniques predominantly probe collinear magnetic configuration and interlayer symmetry-cancellation effects, and therefore naturally reveal layer-parity-dependent responses.

In contrast, THG is a third-order nonlinear optical process that is allowed even in centrosymmetric systems. In the present work, the THG response is phenomenologically associated with the emergence of a chiral spin texture characterized by a finite spin chirality, χ, rather than with net magnetization. Importantly, the proposed chiral spin texture originates from interlayer spin interactions in multilayer $CrPS_4$ and represents a multilayer magnetic effect, accompanied by a slight spin canting within each layer. As such, for multilayer materials, this type of chiral magnetic order is not governed by layer-parity-induced symmetry breaking and is therefore not expected to exhibit a pronounced odd–even layer-number dependence in multilayer $CrPS_4$. Moreover, within our experimental thickness range, the THG_{CD} signal shows a consistent behavior in Fig. R10 (Figure 16 from the Supplementary information), supporting the picture that the observed chiral

magnetic correlations arise from interlayer interactions rather than from finite-size or odd–even layer-number effects. Actually, we have spent a lot of time exfoliating the few-layer $CrPS_4$ using different exfoliating methods. The thinnest flake that we can obtain is 5 nm.

Therefore, the THG_{CD} response in $CrPS_4$ does not necessarily show the odd–even thickness dependence reported in MOKE and SHG studies, but instead reflects the fundamentally different symmetry channels and magnetic degrees of freedom probed by THG compared to MOKE and SHG. We will clarify this distinction and the multilayer effect origin of the chiral spin texture in the revised manuscript.

Manuscript revision:

Line 164-168, page 10. "Within our experimentally accessible thickness range, the THG_{CD} response exhibits consistent behavior with no clear layer-parity dependence. Unlike MOKE and SHG, which are sensitive to net magnetization or layer-parity–dependent symmetry breaking. THG is even allowed in centrosymmetric systems. It suggests that the observed chiral signal is governed primarily by competing near and next-near interlayer exchange interactions that persist in multilayer $CrPS_4$."

Comments from Reviewer 2-3:

3. While the manuscript proposes a non-collinear spiral magnetic ground state, the supporting evidence relies almost entirely on THG_{CD} measurements and phenomenological modeling. Reported in van der Waals magnets, non-collinear magnetic structures have been directly imaged or resolved using techniques such as MOKE, RMCD, and MFM microscopy, which provide spatially resolved information on magnetic domains. The manuscript suggests that THG_{CD} reveals the magnetic ground state of $CrPS_4$. However, THG_{CD} is not a direct magnetic structure determination technique. At present, the claim of a revised magnetic ground state—especially one differing from magnetic structures previously resolved by neutron scattering—remains indirect. If a new magnetic ground state is proposed, more direct experimental evidence for non-collinear magnetism is required.

Response R2-3:

We thank the reviewer for this important comment. We fully agree that THG_{CD} is not a direct magnetic structure determination technique and does not provide real-space imaging of magnetic domains, unlike MOKE, RMCD, or MFM microscopy. Techniques such as magneto-optical Kerr effect (MOKE), reflective magnetic circular dichroism (RMCD), and magnetic force microscopy (MFM) are most sensitive to net magnetization or stray magnetic fields but yield negligible or weak signals from antiferromagnets [Nat. Mater. 24, 226–233 (2025)]. In this work, we propose that the chiral spin texture in $CrPS_4$ is a multilayer effect originating from interlayer spin interactions, accompanied by an additional slight spin canting within each layer. Such a magnetic configuration does not correspond to real-space magnetization textures with strong spatial modulation, such as skyrmions or bubble domains, which exhibit pronounced stray magnetic fields and can be directly imaged by MFM [ACS Nano 18, 20055–20064 (2024)]. Instead, the proposed chiral spin texture involves predominantly antiferromagnetic and weakly canted spins within each layer, resulting in negligible net magnetization and minimal stray fields. Consequently, real-space magnetic imaging techniques such as MFM are not expected to be sensitive to this type of multilayer effect, non-collinear antiferromagnetic order. Furthermore, we have measured the spatially resolved THG_{CD} in Fig. R11 (Figure 14 in the Supplementary information). The finite THG_{CD} is relatively inhomogeneous in the measured area, consistent with the spin spirals across the interlayers' antiferromagnetic order.

Accordingly, we do not claim that THG_{CD} alone uniquely determines the magnetic structure of $CrPS_4$. Instead, our work demonstrates that THG_{CD} provides optical evidence for the presence of non-collinear and chiral magnetic correlations. This allows THG_{CD} to access magnetic information that is complementary to that obtained from linear magneto-optical probes or real-space imaging techniques.

Previous neutron scattering studies primarily resolve long-range, static, and predominantly collinear magnetic order. Weak chiral components, field-induced canting, or non-collinear spiral correlations, especially if spatially averaged or dynamically fluctuating, may remain undetected in such measurements. Moreover, neutron scattering is not suitable for detecting 2D magnets down to atomic thickness and micrometre lateral size [Nat. Mater. 24, 226–233 (2025)]. Our results,

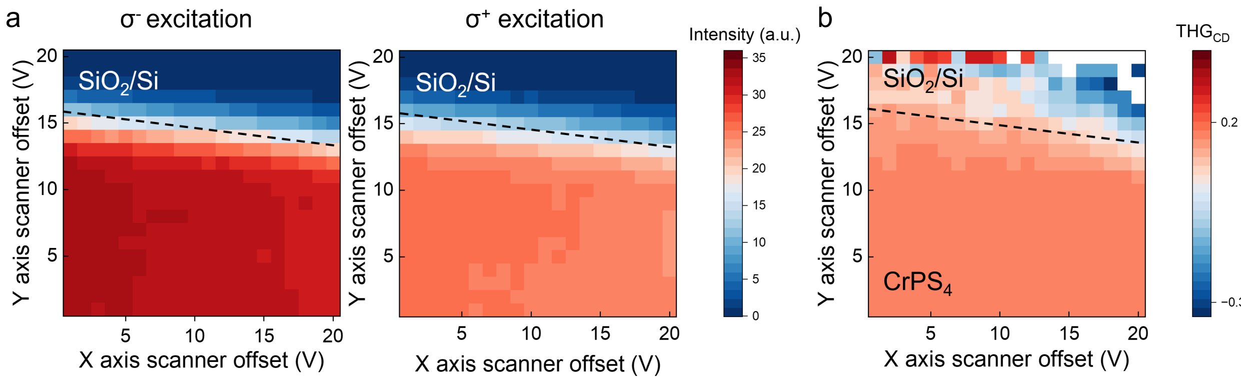

Fig. R11 (Figure 14 of the supplementary information). *THG imaging of the 12 nm thick* $CrPS_4$ *flake at the temperature of 2 K. (a) Spatial mapping of THG intensity excited by left-handed circular polarized* (σ^-) *light and right-handed circular polarized* (σ^+)*. (b) Calculated* THG_{CD} *based on the data from (a).*

therefore, do not contradict existing neutron scattering data but instead suggest the presence of additional non-collinear magnetic correlations beyond the previously reported magnetic structure. We thank the reviewer point out that more direct experimental evidence is required. However, due to the lack of an experimental setup, we cannot perform these and will consider them for future work.

Moreover, to the best of our knowledge, previous reports that use MOKE, RMCD, and MFM and neutron scattering haven't discussed the correlation between the finite spin chirality and their signals. In our work, we emphasize that our conclusions are based on a combined analysis of THG_{CD} measurements and phenomenological modeling in spin chirality, and we regard the proposed chiral spiral state as a physically consistent interpretation. We agree that direct probes of non-collinear magnetism would be highly valuable, and we view our work as motivating future experimental investigations using complementary techniques. Although our developed technology in the work does not directly image the domain, a direct correlation between spin chirality and nonlinear optical responses is definitely desirable, as it would provide a sensitive in situ or near-field probe of complex magnetic structures in low-dimensional magnets.

Manuscript revision:

Line 258-263, page 14. "Although our developed technology in the work does not directly image the domain, establishing a direct correlation between spin chirality and nonlinear optical responses

is highly desirable. To address this, our THG-based approaches have strong potential to serve as sensitive in situ or near-field probes of complex magnetic structures in low-dimensional magnets. For clarity, we compare THG and other commonly used magnetic probes in Supplementary Table 1."

Supplementary information revision:

Line 258-263, page 26. "To the best of our knowledge, previous reports that magneto-optical Kerr effect (MOKE), reflective magnetic circular dichroism (RMCD), magnetic force microscopy (MFM), second harmonic generation (SHG), and neutron scattering techniques haven't discussed the correlation between the finite vector spin chirality and their signals. In our work, we emphasize that our conclusions are based on a combined analysis of THG_{CD} measurements and phenomenological modeling in spin chirality, and we regard the proposed chiral spiral state as a physically consistent interpretation."

Table 1. (Updated Supplementary Table 1) Comparison of different magnetic measurement methods.

Method	Sensitive Quantity	Spatial Resolution	Typical Signal	Detected Structure	References
MOKE	Net magnetization	sub-μm	μrad	FM domains, magnetic bubbles	[7]
RMCD	Net magnetization	μm	$< 1\%$	FM domains, magnetic bubbles	[8]
MFM	Magnetic stray field	nm	None	FM domains, magnetic bubbles	[9]
SHG	Magnetic order	μm	None	AFM domains, magnetic polygraphs	[10]
THG_{CD}	Finite vector spin chirality	μm	$> 10\%$	AFM coplanar spin spirals	This work

Comments from Reviewer 2-4:

4. According to the manuscript, a chiral spiral magnetic structure emerges at low temperature. This would generally be expected to lower the symmetry and potentially break inversion symmetry compared to the high-temperature phase. In this case, a question arises as to whether this symmetry change is reflected in SHG measurements. Whether SHG-CD is enhanced or newly allowed in the low-temperature chiral phase compared to room temperature?

Response R2-4:

We thank the reviewer for this insightful question. While the emergence of a chiral spiral magnetic structure indeed breaks time-reversal symmetry and lowers the magnetic symmetry, it does not necessarily break the crystallographic inversion symmetry of the system. In the present work, the proposed chiral spin texture is a multilayer magnetic effect arising from interlayer spin interactions, without invoking a structural distortion or a global inversion-symmetry breaking of the lattice.

As a result, electric-dipole second-harmonic generation (SHG), which is strictly forbidden in centrosymmetric crystals, is not expected to be activated or significantly enhanced solely due to the emergence of magnetic chirality. Although magnetic-dipole or higher-order SHG contributions may in principle become symmetry-allowed in magnetically ordered phases, these effects are typically much weaker and may remain below our experimental sensitivity. Upgrading the experimental sensitivity of our system will be necessary to assess potential SHG signals in $CrSP_4$, which deserves future investigation.

Comments from Reviewer 2-5:

5. In the temperature-dependent measurements, the THG_{CD} signal does not vanish completely above the Néel temperature. What is the physical origin of the non-zero THG_{CD} signal observed above the Néel temperature?

Response R2-5:

We thank the reviewer for this insightful question. The Néel temperature marks the disappearance of long-range antiferromagnetic order, but it does not imply the complete absence of magnetic correlations. In low-dimensional van der Waals magnets, short-range spin correlations and strong spin fluctuations are known to persist above Néel temperature.

As a higher-order nonlinear optical probe, THG_{CD} is sensitive to higher-order magnetic correlations and can therefore detect contributions from short-range or dynamic spin correlations even in the paramagnetic phase. In addition, under an applied magnetic field, local spin canting or polarization may persist above Néel temperature, giving rise to a weak but finite THG response.

We note that the THG_{CD} signal above Néel temperature is significantly reduced compared

to that in the ordered phase and shows no sharp anomaly, consistent with the absence of long-range magnetic order. The non-zero THG_{CD} observed above Néel temperature might be attributed to short-range magnetic correlations and possible spin fluctuations. We clarify this point in the revised manuscript. And at higher temperatures (e.g., 250 K), the THG_{CD} vanishes completely in Fig. R12 (Figure 18 in the Supplementary information). This behavior is consistent with the thermal destruction of all magnetic correlations at sufficiently high temperatures, leaving only the non-magnetic background contribution, which is negligible in our measurements.

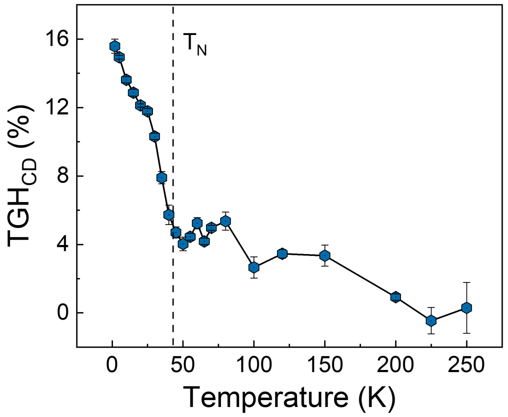

Fig. R12 (Figure 18 of the Updated supplementary information). *THG_{CD} from a ~12.5 nm thick $CrPS_4$ flake measured in the temperature range 2–250 K. T_N: Néel temperature.*

Manuscript revision:

Line 188-192, page 10. "While long-range antiferromagnetic order disappears above the Néel temperature, short-range magnetic correlations and spin fluctuations might persist and contribute to a weak THG_{CD} signal. This residual response is strongly suppressed with increasing temperature and vanishes completely at higher temperatures (e.g., 250 K), consistent with the thermal destruction of all magnetic correlations."

We thank all reviewers for their careful evaluation and constructive comments, which have helped us substantially improve the manuscript. We hope the revised manuscript can now be considered for publication.

The authors

Response letter

Dear Reviewers,

We sincerely thank you for your valuable suggestions and constructive comments. Your feedback has been instrumental in improving the quality and clarity of our manuscript. We are encouraged that both reviewers expressed satisfaction with our previous responses. Reviewer 1 noted that *"I am satisfied with most of the responses..."*, and Reviewer #2 commented that *"The authors have provided a detailed response and revised the manuscript, which has improved the clarity of their arguments..."*.

We are further grateful for your additional comments and for the time and effort devoted to evaluating our work. In the following, we provide a point-by-point response to all comments and have revised the manuscript accordingly. Reviewer comments are shown in *black italics*, our responses are given in blue, and corresponding revisions in the manuscript and Supplementary Information are highlighted in red.

Reviewer #1 (Comments for the Author):

I am satisfied with most of the responses except the following two comments:

We thank the reviewer for the careful evaluation and are pleased that most of the concerns have been addressed. Below, we provide further clarification on the remaining two points.

Comments from Reviewer 1-1-1:

In response R1-8, the authors claim no SHG measured in the spin-canting structure. From symmetry aspect, canted spin order that breaks PT symmetry can generate SHG in electric-dipole approximation. This effect has been predicted (Sci. Adv. 11, eadu6562) and experimentally observed (arXiv:2509.13542) in CrSBr. It is possible that due to the weak SOC, this effect is difficult to observe in SHG. I think the authors should make this point clearer.

Response R1-1-1:

Many thanks for pointing this out. We agree with the reviewer: From a symmetry perspective, the canted spin order could, in principle, allow second-harmonic generation (SHG) within the electric-dipole approximation. The fact that no clear SHG signal is observed from $CrPS_4$ may reflect either an intrinsically weak second-harmonic response or the limited sensitivity of our present setup. As suggested by the reviewer, we have now revised the manuscript.

Manuscript revision:

Line 119-122, page 6: “At the same time, from a symmetry-breaking perspective, the canted spin order can, in principle, induce SHG in the electric-dipole approximation **[42, 57]**. The lack of

an observable SHG signal from $CrPS_4$ may therefore arise either from a weak SHG response or from the limited sensitivity of our current setup."

Comments from Reviewer 1-1-2:

In addition to THG, there are other transport and optical measurements that are sensitive to spin chirality, which I think are closely related to this work and should be cited (Phys. Rev. Lett. 124, 096602, Commun. Phys. 4, 99, Phys. Rev. Lett. 130, 166702, Sci. Adv. 11, eadu6562).

Response R1-1-2:

As suggested by the reviewer, we have cited the works as Refs. 42, 63, 64 and 65 in the main text.

Comments from Reviewer 1-2:

*In response R 1-10, the authors says "The fundamental wavelength used in our experiments (1575 nm, corresponding to a photon energy of around 0.79 eV) lies well below the dominant interband optical transitions of $CrPS_4$, placing the measurement in a non-resonant regime." As in THG, 0.79 eV light can induce resonance even at 3*0.79 eV, I think this is already in the resonant regime.*

Response R1-2:

We thank the reviewer for the comment. We agree with the reviewer. We have carefully reviewed the main text and SI to avoid any use of the term "non-resonant" and prevent potential confusion.

Reviewer #2 (Comments for the Author):

The authors have provided a detailed response and revised the manuscript, which has improved the clarity of their arguments, particularly regarding the magnetic origin of the THG-CD signal and its distinction from SHG and MOKE probes. However, one critical issue raised in my initial review remains insufficiently addressed, and a new contradiction has emerged from their own response.

Many thanks to the reviewer for the careful reading of our revised manuscript and for recognizing the improvements. We also appreciate the reviewer for pointing out the remaining concerns.

Comments from Reviewer 2-1:

1. The authors argue that the observed THG-CD originates from a chiral spin texture driven by competing interlayer exchange interactions—a multilayer magnetic effect. If this were an intrinsic bulk magnetic property, one would expect a clear dependence of the THG-CD signal amplitude on

the number of magnetic layers (thickness), especially when transitioning from a few layers to the multilayer regime. However, the Figure R10 show "within our experimentally accessible thickness range, the THG-CD response exhibits consistent behavior". This lack of thickness sensitivity is paradoxical for a phenomenon claimed to arise from interlayer couplings.

Response R2-1:

We thank the reviewer for the careful reading. We agree with the reviewer. Indeed, a thinner (~5 nm) flake shows a reduced circular dichroism contrast, with THG_{CD} reaching only ~6% at 2 K, as shown in Fig. R1 a and b, while a ~12 nm thick flake features THG_{CD} of ~14%, which is around two times larger than that in the thinner (~5 nm) flake. This behaviour suggests that interlayer interactions indeed play a role in generating the THG_{CD} signal. We have revised the main text to avoid confusion and clarified the thickness difference in the Supplementary Information.

Supplementary information revision:

Line 256-261, page 18: "Specifically, a thinner (~5 nm) flake was also measured and shows a reduced circular dichroism contrast. The THG_{CD} of the thinner (~5 nm) flake reaches only ~6% at 2 K, about half of the ~14% observed in the ~12 nm flake (Figure 16), and becomes negligible at room temperature (297 K), as shown in Figure 17. These results further highlight the important role of interlayer interactions in determining the THG_{CD} response."

Comments from Reviewer 2-2-1:

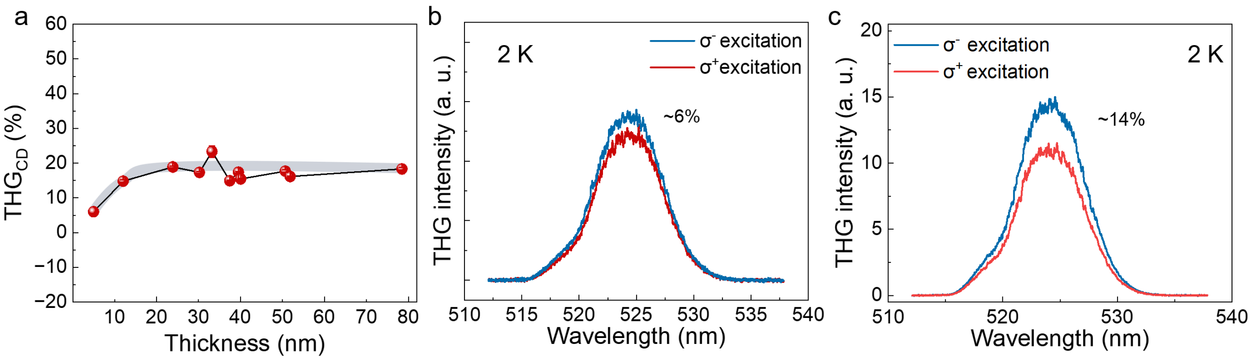

Fig. R1 (Updated from Figure 2b of the main text, Figures 16 and 17 of the supplementary information). Thickness-dependent THG_{CD}. (a) THG_{CD} from $CrPS_4$ with different thicknesses at the temperature of 2 K. THG Spectra obtained from ~5 nm (b) and ~12 nm (c) thin flakes when excited by left- and right-circularly polarized light.

2. While the authors correctly note that techniques like MFM are insensitive to antiferromagnetic textures, the claim of a "non-collinear chiral ground state" still relies entirely on a single optical

probe (THG-CD) and phenomenological modeling. The lack of direct structural evidence remains a significant weakness for a paper claiming the observation of a new magnetic texture.

Response R2-2-1:

We thank the reviewer for this important and thoughtful comment. We agree that our work does not provide a direct real-space or momentum-resolved determination of the magnetic structure. Instead, our work offers a sensitive signature of the average vector spin chirality across the long-range spin spiral phase. We would like to emphasize that obtaining direct experimental observation of antiferromagnetic coplanar spin spirals remains highly challenging, particularly in low-dimensional van der Waals magnets. We also acknowledge that, indeed, combining different state-of-the-art techniques to fully explore complex spin textures in $CrPS_4$ is well deserved for future study.

We have revised the manuscript to clarify this.

Manuscript revision:

Line 264-269, page 14: "We emphasize that THG_{CD} does not directly determine the magnetic structure, and direct structural evidence is still needed to better understand the underlying physics. We also note that previous theoretical studies have shown that spin chirality can appear in transport and optical responses **[42, 63-65]**. Extending such measurements to $CrPS_4$ would therefore be very helpful for understanding the role of chiral magnetic correlations in this system. Nevertheless, our results suggest that THG-based techniques could be a useful way to probe complex magnetic states."

Comments from Reviewer 2-2-2:

I cannot recommend publication in the current state and I hope the author will adequately address the above issues in the next revision.

Response R2-2-2:

We hope that the revisions and clarifications provided in the current version adequately address the reviewer's concerns and meet the requirements for publication.

We thank all reviewers again for their careful evaluation and constructive comments, which have helped us substantially improve the manuscript. We hope the revised manuscript can now be considered for publication.

The authors

Response letter

Dear Reviewers,

We would like to express our sincere gratitude to the reviewer for their time and the constructive feedback provided on our manuscript. We are particularly encouraged by the positive decision to accept our revised manuscript in principle with all your help.

Following your insightful suggestions, we have carefully revised the manuscript to address each point raised.

REVIEWERS' COMMENTS
Reviewer #2 (Remarks to the Author):
The authors have responded carefully to the issues I raised. Specifically, they have made descriptive corrections regarding the thickness-dependent data. In addition to the THG measurements, the authors also performed SHG measurements on CrPS4, but no SHG signal was detected. However, both theoretical and experimental studies indicate that symmetry breaking (either lattice or magnetic) in CrPS4 can give rise to large SHG signals (e.g., Adv. Optical Mater. 2024, 2400943). In these related studies, a pronounced thickness dependence was also observed, which aligns well with the authors' analysis of the origins of the SHG and THG signals. I recommend that the authors further compare this part of their work with the existing literature to ensure an accurate and appropriate description.

Response:

We thank the reviewer for the careful evaluation of our manuscript and for bringing this relevant reference to our attention. We agree that the study reported in Adv. Optical Mater. 2024, 2400943 provides valuable insights into SHG in $CrPS_4$, including its thickness dependence.

However, the primary focus of our work is the chiral third-order nonlinear optical response rather than the conventional SHG response itself. Since the results and mechanisms discussed in the reference are beyond the main scope of the present study, we have not included a comparison in the revised manuscript. Nevertheless, to acknowledge this relevant prior work and provide broader context for nonlinear optical studies of $CrPS_4$, we have cited it in the main text as Ref. 59.

We thank the reviewer once again and sincerely appreciate their constructive comments and suggestions, which have greatly helped us to improve the clarity and quality of the manuscript.

The authors
